# The space–time structure of hadronization in the Lund model

**DOI:** 10.1140/epjc/s10052-018-6459-8

**Published:** 2018-11-28

**Authors:** Silvia Ferreres-Solé, Torbjörn Sjöstrand

**Affiliations:** 1 Present Address: NIKHEF, Science Park 105, 1098 XG Amsterdam, The Netherlands; 2 Theoretical Particle Physics, Department of Astronomy and Theoretical Physics, Lund University, Sölvegatan 14A, 223 62 Lund, Sweden

## Abstract

The assumption of linear confinement leads to a proportionality of the energy–momentum and space–time pictures of fragmentation for a simple $\mathrm{q}\bar{\mathrm{q}}$ system in the Lund string model. The hadronization of more complicated systems is more difficult to describe, and in the past only the energy–momentum picture has been implemented. In this article also the space–time picture is worked out, for open and closed multiparton topologies, for junction systems, and for massive quarks. Some first results are presented, for toy systems but in particular for LHC events. The density of hadron production is quantified under different conditions. The (not unexpected) conclusion is that this density can become quite high, and thereby motivate the observed collective behaviour in high-multiplicity $\mathrm{p}\mathrm{p}$ collisions. The new framework, made available as part of the Pythia event generator, offers a starting point for future model building in a number of respects, such as hadronic rescattering.

## Introduction

The Standard Model of particle physics is solidly established by now, and has been very successful in describing all perturbatively calculable observables for LHC $\mathrm{p}\mathrm{p}$ collisions, i.e. those dominated by large momentum transfer scales [1]. But at lower scales the perturbative approach breaks down, and phenomenological models have to be developed.

One of the underlying assumptions for these models has been that the nonperturbative hadronization process, wherein the perturbatively produced partons turn into observable hadrons, is of a universal character. Then relevant nonperturbative parameters can be determined e.g. from LEP data, and afterwards be applied unmodified to LHC $\mathrm{p}\mathrm{p}$ collisions. The hadronizing partonic state is quite different in the two processes, however. Firstly, the composite nature of the incoming protons leads to multiple semiperturbative parton–parton collisions, so-called MultiParton Interactions (MPIs) [2, 3], and also to beam remnants and initial-state QCD radiation. Secondly, the high number of interacting partons leads to the possibility of nontrivial and dynamically evolving colour topologies, collectively referred to as Colour Reconnection (CR) phenomena. Both MPIs and CR need to be modelled, and involve further new parameters. (CR has been observed in the cleaner $\mathrm{e}^+ \mathrm{e}^- \rightarrow \mathrm{W}^+ \mathrm{W}^-$ process by the LEP collaborations [4], but that information is not easily transposed to the $\mathrm{p}\mathrm{p}$ context.)

The most successful approach to providing a combined description of all relevant phenomena, at all scales, is that of event generators. Here Monte Carlo methods are used to emulate the quantum mechanical event-by-event fluctuations at the many stages of the evolution of an event [5]. For $\mathrm{p}\mathrm{p}$ physics the three most commonly used generators are Pythia [6, 7], Herwig [8, 9] and Sherpa [10]. Fragmentation here proceeds either via strings [11], for the former, or via clusters [12], for the latter two. A note on terminology: “fragmentation” and “hadronization” can be used almost interchangeably, but the former is more specific to the breakup of a partonic system into a set of primary hadrons, whereas the latter is more generic and can also include e.g. decays of short-lived resonances.

In spite of an overall reasonable description, glaring discrepancies between data and models have been found in some cases. Most interesting is that high-multiplicity LHC $\mathrm{p}\mathrm{p}$ events show a behaviour that resembles the one normally associated with heavy-ion collisions and the formation of a Quark-Gluon Plasma (QGP). In particular, ALICE has shown that the fraction of strange baryons increases with multiplicity, the more steeply the more strange quarks the baryon contains, while the proton rate is not enhanced [13]. Long-range azimuthal “ridge” correlations have also been observed by both CMS [14, 15] and ATLAS [16], as well as other signals of collective flow [17–19].

This is unlike conventional expectations, that QGP formation requires volumes and timescales larger than the one that can be obtained in $\mathrm{p}\mathrm{p}$ collisions [20–22]. Nevertheless core–corona models have been developed, like the one implemented in EPOS [23], where a central high-density region can turn into a QGP, while the rest of the system remains as normal individual strings. Other mechanisms that have been proposed include rope formation [24] and shoving [25], or an environment-dependent string tension [26]. Common for all of them is that they introduce a space–time picture of the collision process.

In the traditional Lund string model [11] the linear confinement potential leads to a linear relationship between the energy–momentum and space–time pictures of a simple $\mathrm{q}\bar{\mathrm{q}}$ fragmenting system. Many of the above models are based on the approximation of a number of such simple strings, parallel along the $\mathrm{p}\mathrm{p}$ collision axis but displaced in the transverse plane by the collision/MPI geometry.

For a generic multiparton system, like $\mathrm{q}\mathrm{g}_1\mathrm{g}_2\ldots \mathrm{g}_n\bar{\mathrm{q}}$, only an energy–momentum picture has been available until now [27]. The purpose of this article is to overcome that limitation, and provide a full space–time picture of the hadronization process, as part of the Pythia event generator.^1^ This will offer a natural starting point for more detailed future studies of a number of collective effects. The models mentioned above deal with the space–time structure before (like core–corona or shove) or during (like ropes or QGP) fragmentation. To this we would also add a possibility for studies of what happens after the first stages of the hadronization, when hadronic rescattering and decays can occur in parallel. In addition to the already mentioned observables, Bose–Einstein correlations could also be used to characterize final states.

A warning is that we are applying semiclassical models to describe the quantum world. Formally the Heisenberg uncertainty relations impose limits on how much simultaneous energy–momentum and space–time information one can have on an individual hadron. Our approach should still make sense when averaged over many hadrons in many events, as will always be the case.

The plan of the article is as follows. Section 2 gives a brief summary of relevant earlier work, on the “complete” description of the simple $\mathrm{q}\bar{\mathrm{q}}$ system [11], and on the energy–momentum picture of an arbitrary partonic system [27]. Section 3 then introduces the new framework that provides a space–time picture also in a general configuration. Several special cases need to be addressed, and technical complications have to be sorted out, with some details relegated to two appendices. Section 4 contains some first studies, partly for toy systems but mainly for LHC events. This is without any of the collective effects that may be added later, but still provides an interesting overview of the overall space–time evolution of hadronization at the LHC. Finally, Sect. 5 concludes with a summary and outlook.

Natural units are assumed throughout the article, i.e. $c = \hbar = 1$. By default energy, momentum and mass is given in GeV, and space and time in fm.

**Fig. 1:**
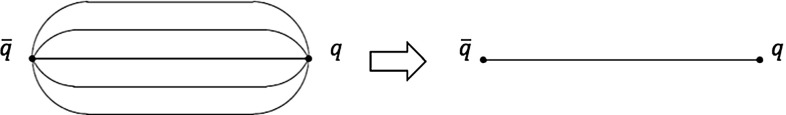
A simplified colour-field topology in a $\mathrm{q}\bar{\mathrm{q}}$ system and its further simplified string representation

## The Lund String model

### The linear force field in QCD

Confinement is one of the most fundamental properties of QCD. It can be viewed as a consequence of an approximately linear term in the QCD potential,

1 $$\begin{aligned} V_{\mathrm {QCD}}(r) \approx -\frac{4}{3} \, \frac{\alpha _{\mathrm{s}}}{r} + \kappa \, r, \end{aligned}$$

between a quark and an antiquark in an overall colour singlet state, where *r* is the distance between them and $\alpha _{\mathrm{s}}$ is the strong coupling constant. The presence of a linear term was first inferred from hadron spectroscopy (Regge trajectories), from which a $\kappa \approx 1$ GeV/fm can be extracted, and has later been confirmed by lattice QCD calculations.

The linear term dominates at large distances, and in the Lund string model only this term is used to describe the breakup of a high-mass $\mathrm{q}\bar{\mathrm{q}}$ system into several smaller-mass ones. Then the full colour field can be approximated by a one-dimensional string stretched straight between the $\mathrm{q}$ and $\bar{\mathrm{q}}$, Fig. 1. This string can be viewed as parametrizing the center of a cylindrical region of uniform width along its full length, such that the longitudinal and transverse degrees of freedom almost completely decouple.

### The two-parton system

The Lund model is easiest to understand in the context of a simple quark–antiquark pair created at the origin (e.g. by $\mathrm{e}^+ \mathrm{e}^-$ annihilation) and moving out along the $\pm z$ axis. Neglecting the transverse degrees of freedom, the Hamiltonian can then be written as [11]

2 $$\begin{aligned} H = E_{\mathrm{q}} +E_{\bar{\mathrm{q}}} + \kappa |z_{\mathrm{q}}-z_{\bar{\mathrm{q}}}| . \end{aligned}$$

Here $|z_{\mathrm{q}}-z_{\bar{\mathrm{q}}}|$ is the distance between $\mathrm{q}$ and $\bar{\mathrm{q}}$, and $E_{\mathrm{q}}$ and $E_{\bar{\mathrm{q}}}$ are the energies of the $\mathrm{q}$ and $\bar{\mathrm{q}}$. With both assumed massless, it also holds that $E_{\mathrm{q}/\bar{\mathrm{q}}}= |\mathbf {p}_{\mathrm{q}/\bar{\mathrm{q}}}|=|p_{z,\mathrm{q}/\bar{\mathrm{q}}}|$.

From the Hamiltonian, the equation of motion gives rise to a linear relation between the space–time and the energy–momentum pictures,

3 $$\begin{aligned} \left| \frac{\mathrm{d}p_{z,\mathrm{q}/\bar{\mathrm{q}}}}{\mathrm{d}t} \right| = \left| \frac{\mathrm{d}p_{z,\mathrm{q}/\bar{\mathrm{q}}}}{\mathrm{d}z} \right| = \left| \frac{\mathrm{d}E_{\mathrm{q}/\bar{\mathrm{q}}}}{\mathrm{d}t} \right| = \left| \frac{\mathrm{d}E_{\mathrm{q}/\bar{\mathrm{q}}}}{\mathrm{d}z} \right| = \kappa . \end{aligned}$$

The signs of the derivatives depend both on the direction of motion of the parton and on the direction the string pulls it in. When the parton moves out along the $+z$ axis, e.g., the string pulls the parton in the $-z$ direction, and all signs are negative.

**Fig. 2:**
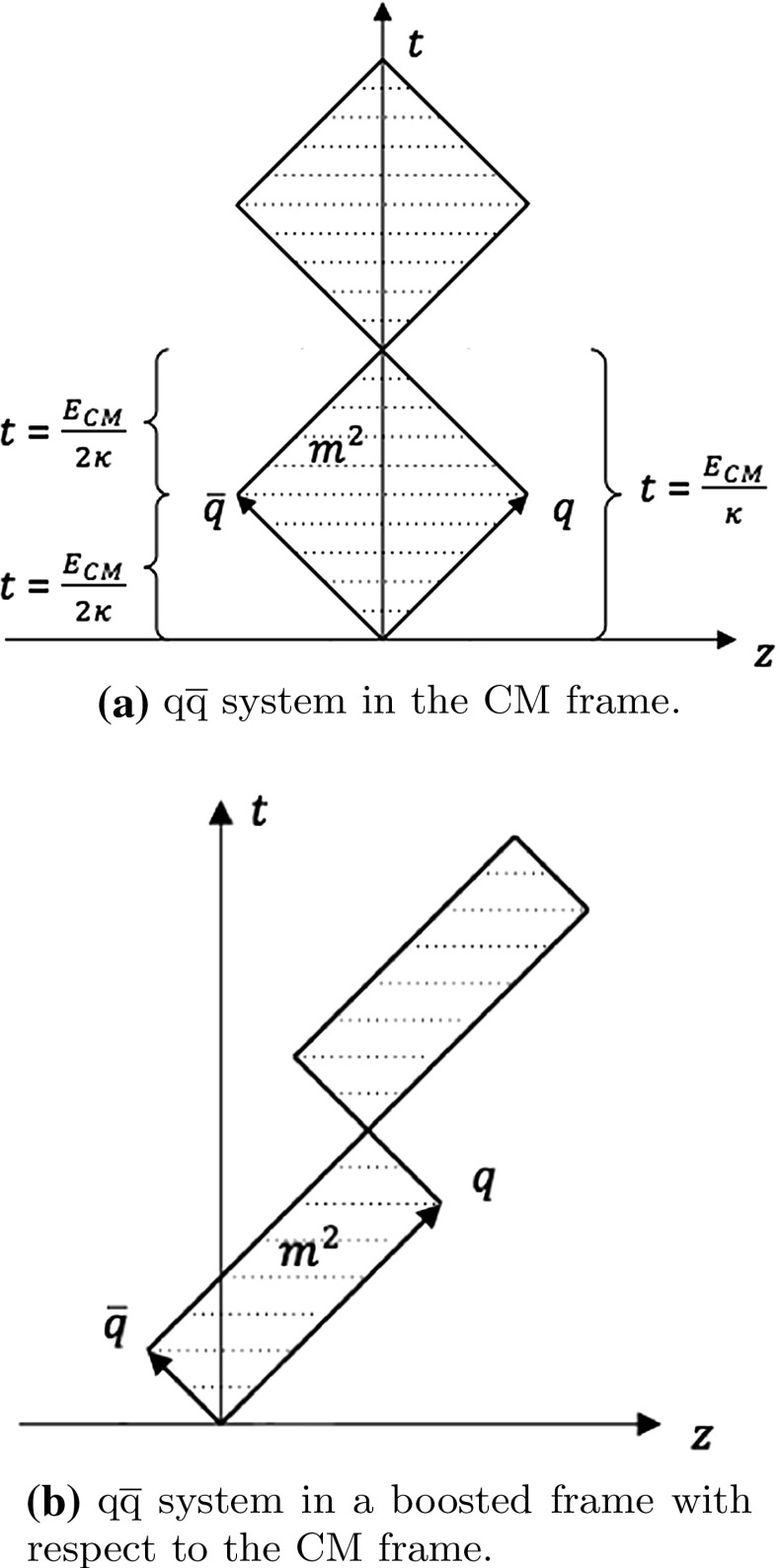
The motion of a $\mathrm{q}\bar{\mathrm{q}}$ system, with massless $\mathrm{q}$ and $\bar{\mathrm{q}}$

#### Simple string motion

In the absence of string breaks, the motion of the simple $\mathrm{q}\bar{\mathrm{q}}$ system in its rest frame can be described as a “yo-yo” motion, where a string is alternatingly “reeled out” and “reeled in”, Fig. 2a. In the first quarter of a period the $\mathrm{q}$ and $\bar{\mathrm{q}}$ are moving apart from each other with the speed of light, $z = \pm t$, such that the string length $l_{\mathrm {string}} = 2t$. Therefore, the four-momenta of the $\mathrm{q}$, $\bar{\mathrm{q}}$ and the string evolve with time as

4 $$\begin{aligned} p_{\mathrm{q}/\bar{\mathrm{q}}}(t)= & {} \left( \frac{E_{\mathrm{cm}}}{2}-\kappa t \right) \, (1; 0, 0, \pm 1) ,\nonumber \\ p_{\mathrm {string}}(t)= & {} (2\kappa t; 0, 0, 0) , \end{aligned}$$

where $E_{\mathrm{cm}}$ is the center-of-mass energy of the full system. At time $t = E_{\mathrm{cm}}/2\kappa$ all the energy is carried by the string, whose string tension then forces the $\mathrm{q}$ and $\bar{\mathrm{q}}$ to turn around. In the second quarter of the period the string length decreases like $l_{\mathrm {string}} = 2(E_{\mathrm{cm}}/\kappa - t)$, and energy and momentum is transferred back to the $\mathrm{q}/\bar{\mathrm{q}}$. At $t = E_{\mathrm{cm}}/\kappa$ the string has vanished and the $\mathrm{q}/\bar{\mathrm{q}}$ are back at the origin, but now moving in the ${\mp } z$ direction. The second half of the full period therefore becomes a repeat of the first half, only with the role of $\mathrm{q}$ and ${\bar{\mathrm{q}}}$ interchanged. Normally string breaks are assumed to occur so rapidly that only the first quarter of the first period needs to be considered.

The kinematics of the yo-yo motion can conveniently be rewritten in terms of light-cone coordinates, both in energy–momentum, $\tilde{p}^{\pm } = E {\pm } p_{z}$, and in space–time, $\tilde{z}^{\pm } = t {\pm } z$. For instance, for the quark in the first quarter period, $\tilde{z}_{\mathrm{q}}^- = \tilde{p}_{\mathrm{q}}^- = 0$, $\tilde{z}_{\mathrm{q}}^+ = 2t$, $\tilde{p}_{\mathrm{q}}^+ = E_{\mathrm{cm}}- 2 \kappa t = E_{\mathrm{cm}}- \kappa \tilde{z}_{\mathrm{q}}^+$, such that $\mathrm{d}\tilde{p}_{\mathrm{q}}^+/\mathrm{d}\tilde{z}_{\mathrm{q}}^+ = -\kappa$. $\tilde{p}^{\pm }$ also obey the relation

5 $$\begin{aligned} \tilde{p}^+ \tilde{p}^-= & {} (E+p_{z}) (E-p_{z}) = m^2 + p_x^2 + p_y^2\nonumber \\= & {} m^2 + p_{\perp }^2 = m_{\perp }^2 , \end{aligned}$$

which reduces to $\tilde{p}^+ \tilde{p}^- = m^2$ when $p_x = p_y = 0$.

The simplest yo-yo system can be generalized as illustrated in Fig. 2b, where the quark and the antiquark have different initial energies, $E_{\mathrm{q}} \ne E_{\bar{\mathrm{q}}}$. Equivalently, this system can be viewed as a boosted copy of the rest-frame setup in Fig. 2a. The energy–momentum and space–time coordinates suffer simultaneous transformations under a longitudinal boost, and Eq. (3) holds also after the boost. The transformation is especially easily formulated in light-cone coordinates, where $\tilde{p}'^{\pm } = k^{\pm 1} \tilde{p}^{\pm }$ with $k = \sqrt{(1 + \beta )/(1 - \beta )}$ for a boost with velocity $\beta$, and similarly for $\tilde{z}^{\pm }$.

Note that a string piece with $E = \kappa l$ but $p_z = 0$ in the original rest frame will obtain a $p_z \ne 0$ after a boost to the frame with $E_{\mathrm{q}} \ne E_{\bar{\mathrm{q}}}$. This is in seeming contradiction with a description set up in a rest frame where $E_{\mathrm{q}} \ne E_{\bar{\mathrm{q}}}$ from the onset, where one would again expect $p_z = 0$. The solution is that a string piece is an extended object, so that the two ends of it, if originally simultaneous, will no longer be it after the boost. Only a string piece at constant time in the new frame will obey $E = \kappa l$ and $p_z = 0$ there.

#### String breaking and hadron formation

As described in the previous section, the potential energy stored in the string increases with the separation between an original $\mathrm{q}_0$ and $\bar{\mathrm{q}}_0$. This makes the creation of a new $\mathrm{q}_1\bar{\mathrm{q}}_1$ pair in the string energetically favourable, if the invariant mass of the system is big enough. It is here assumed that colours are matched so that the original colour-singlet $\mathrm{q}_0\bar{\mathrm{q}}_0$ string breaks into two pieces, $\mathrm{q}_0\bar{\mathrm{q}}_1$ and $\mathrm{q}_1\bar{\mathrm{q}}_0$, that separately are colour singlets. By local flavour conservation the $\mathrm{q}_1$ and $\bar{\mathrm{q}}_1$ have to be created in the same vertex. They are created with vanishing energy, and are then pulled apart by the string tension. Naively, the probability for the string to break increases with time, because the string length increases. On the other hand, a break can inhibit later breaks, since each break fragments the string into two smaller systems, leaving an in-between region without a string. If on-mass-shell criteria for hadrons are ignored, as in the Artru–Mennessier model [29], a naive constant breakup probability per unit string area then is modified by an exponential-decay factor.

**Fig. 3:**
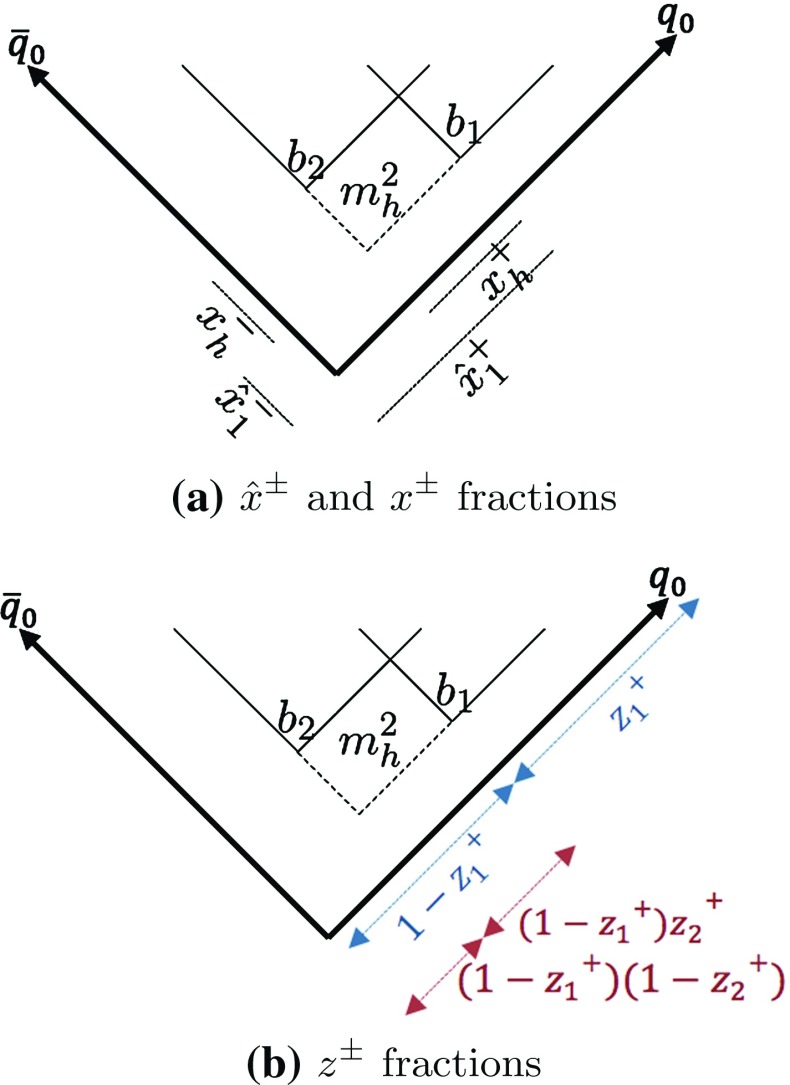
Simple $\mathrm{q}\bar{\mathrm{q}}$ system, where $\mathrm{q}$ and $\bar{\mathrm{q}}$ are massless, with two breaks, $b_1$ and $b_2$. The light-cone coordinates are normalized to unity

In general, several string breaks can occur between the $\mathrm{q}_0$ and $\bar{\mathrm{q}}_0$. Consider two adjacent ones, $b_1$ at $(t_1, z_1)$ and $b_2$ at $(t_2, z_2)$, as depicted in Fig. 3. The $\mathrm{q}_1$ from the $b_1$ vertex combines with the $\bar{\mathrm{q}}_2$ from the $b_2$ vertex, forming a hadron $\mathrm{q}_1\bar{\mathrm{q}}_2$ with mass $m_h$. Since $\mathrm{q}_1$ and $\bar{\mathrm{q}}_2$ are created with no energy–momentum, the four-momentum of the hadron entirely comes from the intervening string piece, which can be read off like [11]

6 $$\begin{aligned} E_h= & {} \kappa (z_1-z_2) ,~~~ p_{zh} = \kappa (t_1-t_2) ,\nonumber \\ \tilde{p}_h^{\pm }= & {} \kappa \left| \tilde{z}_1^{\pm } - \tilde{z}_2^{\pm } \right| . \end{aligned}$$

Next consider the quantities $\hat{x}^{\pm }$ and $x^{\pm }$, illustrated in Fig. 3a. The former represent the light-cone coordinates of breakup vertices, scaled by the corresponding coordinates of the $\mathrm{q}_0$ and $\bar{\mathrm{q}}_0$ turning points, so as to be restricted to a physical region $0 \le x^{\pm } \le 1$. The latter represents the light-cone separation between two (adjacent) breaks, correspondingly scaled. For the $\mathrm{q}_1\bar{\mathrm{q}}_2$ hadron this translates to $x_h^+ = \hat{x}_1^+ - \hat{x}_2^+$, $x_h^- = \hat{x}_2^- - \hat{x}_1^-$. Defining the two four-vectors $p^+ = p_{\mathrm{q}_0}(t=0) = E_{\mathrm{q}_0}(1; 0, 0, 1)$ and $p^- = p_{\bar{\mathrm{q}}_0}(t=0) = E_{\bar{\mathrm{q}}_0}(1; 0, 0, -1)$, and using the proportionality between space–time and energy–momentum, the hadron four-momentum then becomes

7 $$\begin{aligned} p_h = x_h^+ \, p^+ + x_h^- \, p^- . \end{aligned}$$

Although Eq. (7) has been derived for a system in which $\mathrm{q}$ and $\bar{\mathrm{q}}$ are moving in opposite directions, it is valid in all frames, which makes the $\hat{x}^{\pm }$ coordinates and $x^{\pm }$ fractions most useful. Since $E_{\mathrm{cm}}^2 = (p^+ + p^-)^2 = 2p^+p^-$, the hadron mass obeys

8 $$\begin{aligned} m_h^2= & {} p^{2}_h= \left( x_h^+ \, p^+ + x_h^- \, p^- \right) ^2\nonumber \\= & {} x_h^+x_h^- \, 2 p^+ p^- = x_h^+x_h^- \, E_{\mathrm{cm}}^2. \end{aligned}$$

Do note the factor of 2 for the $p^{\pm }$ vectors in $E_{\mathrm{cm}}^2 = 2p^+p^-$, as opposed to the relation $E_{\mathrm{cm}}^2 = \tilde{p}^+ \tilde{p}^-$ for the two scalar quantities $\tilde{p}^{\pm }$, and correspondingly for the hadronic subsystems.

Each breakup vertex is characterized by its invariant time $\tau$. A convenient corresponding energy–momentum quantity is

9 $$\begin{aligned} \varGamma = (\kappa \tau ) ^2 = \kappa ^2 \left( t^2 - x^2 -y^2 -z^2\right) . \end{aligned}$$

which geometrically corresponds to the string area in the backwards light cone of the vertex. Using the notation of Fig. 3a it can also be expressed as

10 $$\begin{aligned} \varGamma = \left( \hat{x}^+ \, p^+ + \hat{x}^-\, p^-\right) ^2 = \hat{x}^+ \, \hat{x}^- \, E_{\mathrm{cm}}^2. \end{aligned}$$

#### Selection of breakup vertices

The breakup vertices are causally disconnected. That is, $b_1$ and $b_2$ in Fig. 3 have a spacelike separation. Which happens first then depends on the Lorentz frame in which the event is studied. It is therefore possible to describe the fragmentation process starting from the hadron closest to the $\mathrm{q}_0$ end and then moving towards the $\bar{\mathrm{q}}_0$ one, or the other way around. Assuming e.g. that $b_1$ has already been selected, so that $\hat{x}_1^{\pm }$ are fixed, then the selection of the two $x_h^{\pm }$ values of the hadron defines the location of $b_2$. But, assuming that the hadron and its mass are already specified, the mass constraint in Eq. (8) reduces it to one degree of freedom. For the fragmentation from the $\mathrm{q}_0$ side this is conveniently chosen to be the $x_h^+$ values. More specifically, $z^+$ fractions are introduced, as illustrated in Fig. 3b, as the hadron fraction of whatever system light-cone momentum that still remains after the production of previous hadrons. That is, the first hadron $\mathrm{q}_0 \bar{\mathrm{q}}_1$ acquires a fraction $z_1^+ = x^+_1$ of the total $\tilde{p}^+$ of the system, while the remnant-system is left with a $\tilde{p}^+$ fraction of $1 - z_1^+ = 1 - x_1^+$. The second hadron $\mathrm{q}_1\bar{\mathrm{q}}_2$ takes a fraction $z_2^+$ from the leftover $\tilde{p}^+$, i.e. $x_2^+ = z_2^+ \, (1-z_1^+)$, leaving a new remainder fraction $(1-z_1^+)(1-z_2^+)$. Since the fragmentation process is iterative, the $x^{\pm }$ fractions related to hadron *i* can be written as

11 $$\begin{aligned}&x_i^+ = z_i^+ \, \prod _{j=1}^{i-1} \left( 1-z_{j}^+\right) , \nonumber \\&x_i^-= \frac{m_i^2}{x_i^+ \, E_{\mathrm{cm}}^2} , \end{aligned}$$

where the relation for $x_i^-$ is given by Eq. (8).

Alternatively the fragmentation could have been described from the $\bar{\mathrm{q}}_0$ end in terms of negative light-cone fractions $z^-$ and $x^-$. Since the breakup points are causally disconnected, the two procedures should result in the same average particle distribution. This requirement, “left–right symmetry”, gives a probability distribution [11, 30]

12 $$\begin{aligned} f(z) \propto \frac{(1-z)^a}{z} \, \exp \left( -b\frac{m_h^2}{z} \right) , \end{aligned}$$

for the *z* value of each new hadron, where $z = z^+$ ($z = z^-$) for fragmentation from the $\mathrm{q}_0$ ($\bar{\mathrm{q}}_0$) end. The *a* and *b* are parameters that should be tuned to reproduce the experimental data. Hence, *f*(*z*) determines how the individual vertices correlate in order to create a hadron of mass $m_h$ by taking a fraction *z* of the energy–momentum left in the system. Note that the form of *f*(*z*) does not depend on previous steps taken, which leads to a flat rapidity plateau of the inclusive hadron production.

The $\varGamma$ values of breakup vertices can be obtained recursively by simple geometrical considerations,

13 $$\begin{aligned} \varGamma _i = (1-z_i) \left( \varGamma _{i-1} + \frac{m_i^2}{z_i} \right) , \end{aligned}$$

where $\varGamma _i$ and $\varGamma _{i-1}$ are the scaled squared invariant times of the *i* and $i-1$ breakups, respectively. The $\mathrm{q}_0$ and $\bar{\mathrm{q}}_0$ turning points define $\varGamma _0 = 0$. The inclusive $\varGamma$ distribution, after some steps away from the endpoint(s), is

14 $$\begin{aligned} P(\varGamma ) \propto \varGamma ^a \, \exp {(-b\varGamma )} \end{aligned}$$

with the same *a* and *b* as in Eq. (12).

**Fig. 4:**
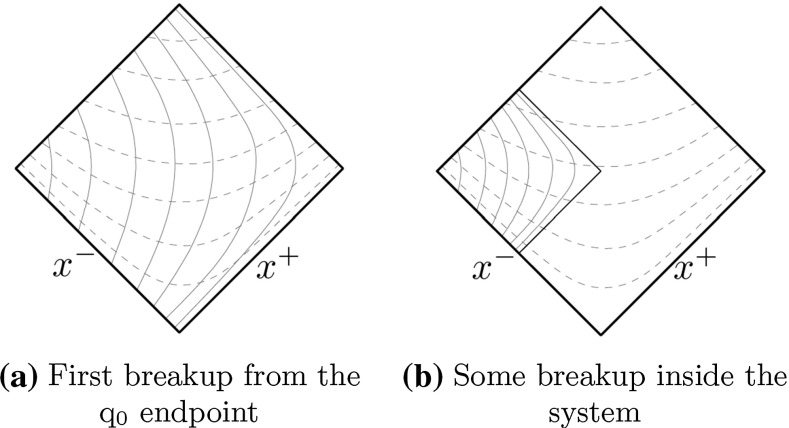
Hyperbolae of constant $\varGamma$ and $m_h^2$ represented by dashed and full lines, respectively

The breaks of the string can be determined from Eq. (11) by iteratively picking $z_i$ values according to Eq. (12) for the hadrons with masses $m_i$ . This works well for the simple $\mathrm{q}_0 \bar{\mathrm{q}}_0$ system, but Eq. (11) will not hold in systems with more than two partons. To this end an alternative procedure can be introduced [27] via $\varGamma$ recursion. Here a $z = z_i$ is still selected by Eq. (12), and converted to a $\varGamma _i$ by Eq. (13). As illustrated in Fig. 4, each fixed $\varGamma$ value corresponds to a hyperbola with the origin as its center. Correspondingly each fixed $m_i$ corresponds to a hyperbola with the $i-1$ vertex as its center. Therefore a given $(m_i, \varGamma _i)$ pair corresponds to the unique crossing of two hyperbolae at the location of the next vertex.

#### The tunneling process

Up to this point, we have assumed that the $\mathrm{q}_i\bar{\mathrm{q}}_i$ pairs generated from string breakings are massless and have no transverse momenta. Both $\mathrm{q}_i$ and $\bar{\mathrm{q}}_i$ are then created as real particles at a common space–time location, with vanishing energy–momentum. If the pair is massive or carries transverse momentum, the $\mathrm{q}_i$ and $\bar{\mathrm{q}}_i$ still have to be created in the same space–time location, but as virtual particles. Each now has to tunnel out a distance $l = m_{\perp }/\kappa$ to acquire enough energy from the string to correspond to its transverse mass $m_{\perp }$. This tunneling results in a Gaussian suppression factor

15 $$\begin{aligned} \exp \left( - \frac{\pi m_{\perp }^2}{\kappa } \right) = \exp \left( -\frac{\pi m^2}{\kappa } \right) \exp \left( -\frac{\pi p_{\perp }^2}{\kappa } \right) . \end{aligned}$$

A consequence of this mechanism is the suppression of heavy quark production in string breaks, approximately like $\mathrm{u}\bar{\mathrm{u}}: \mathrm{d}\bar{\mathrm{d}}: \mathrm{s}\bar{\mathrm{s}}: \mathrm{c}\bar{\mathrm{c}}\approx 1 : 1 : 0.3 : 10^{-11}$ [11]. It is therefore assumed that $\mathrm{c}$ and $\mathrm{b}$ production only occurs by perturbative processes.

The combination of a $\mathrm{q}_{i-1}$ and a $\bar{\mathrm{q}}_i$ gives the flavour of a meson but does not fully specify it. The quark spins can combine e.g. to produce a pseudoscalar or a vector meson, and flavour-diagonal mesons mix, and so on. All of these aspects are relevant for the model as a whole, but for the considerations in this article we only need to know the masses of the produced mesons. Similarly for baryon production, where the production mechanisms are less well understood, whether “diquark” or “popcorn” [31, 32]. In the latter approach actually three different production vertices are involved, one for each of the quarks, but also here an effective description in terms of two, as for mesons, is meaningful. A diquark is taken to be a colour antitriplet, just like an antiquark, and we thus use the notation $\bar{\mathrm{q}}$ as shorthand for either of them.

Since the string itself has no transverse motion, it is assumed that the transverse momentum is locally compensated inside each $\mathrm{q}_i\bar{\mathrm{q}}_i$ pair. The transverse momentum of a hadron $\mathrm{q}_{i-1}\bar{\mathrm{q}}_i$ is then given by the vector sum of its constituent transverse momenta. The hadron masses in Sect. 2.2.2 have to be replaced by the corresponding transverse masses.

#### Massive quarks

Although massive quarks are not created from string breaking, they can be generated in the hard process and form a system that might fragment further. In this section, the yo-yo model is extended to account for massive quarks as the endpoints of the system. Since the massive $\mathrm{q}$ and $\bar{\mathrm{q}}$ do not travel at the speed of light, their motion is described by hyperbolae instead of straight lines.

**Fig. 5:**
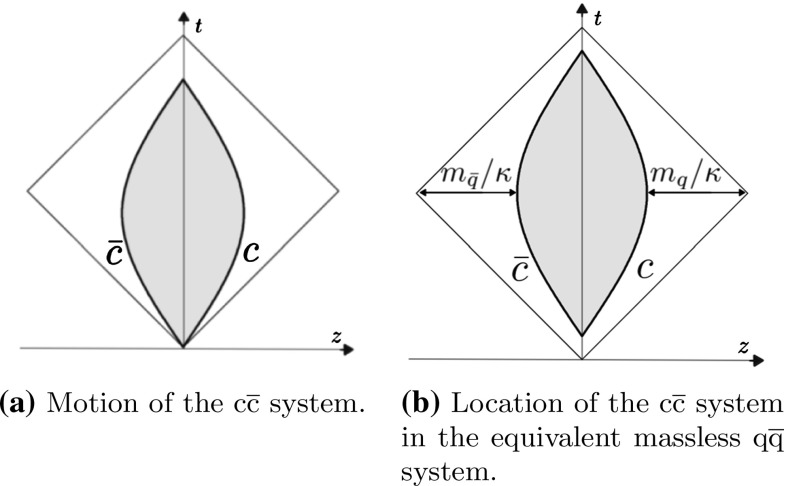
The $c\bar{c}$ system and the equivalent system formed by massless $\mathrm{q}$ and $\bar{\mathrm{q}}$

To study the motion of the massive yo-yo system, consider a $\mathrm{c}\bar{\mathrm{c}}$ system in the CM frame, in which $\mathrm{c}$ and $\bar{\mathrm{c}}$ are moving along the *z* axis in opposite directions. The massive yo-yo system is depicted in Fig. 5a, along with the massless case for comparison. At time $t = 0$,

16 $$\begin{aligned} E_{\mathrm{c}}(0) = E_0 = \frac{E_{\mathrm{cm}}}{2} ,~~~ p_{z,\mathrm{c}}(0) = p_0 = \sqrt{E_0^2 - m_{\mathrm{c}}^2} . \end{aligned}$$

The proper relativistic definition of force, $\mathrm{d}p_{z}/\mathrm{d}t = \pm \kappa$, then gives

17 $$\begin{aligned} p_{z,\mathrm{c}}(t)= & {} p_0 - \kappa t ,\nonumber \\ E_{\mathrm{c}}(t)= & {} \sqrt{p_{z,\mathrm{c}}^2(t) + m_c^2} ,\nonumber \\ z_{\mathrm{c}}(t)= & {} \frac{E_0 - E_{\mathrm{c}}(t)}{\kappa } , \end{aligned}$$

with the motion of the $\bar{\mathrm{c}}$ its mirror image. Notice that the oscillation time is reduced by a factor $p_0/E_0$ relative to the massless system with the same $E_0$.

**Fig. 6:**
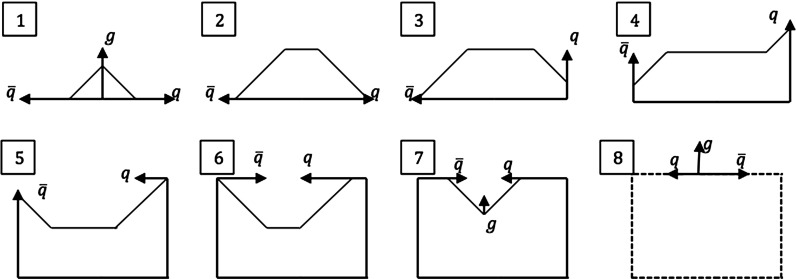
Time evolution of the $\mathrm{q}\mathrm{g}\bar{\mathrm{q}}$ system formed by massless partons in a frame where the gluon moves in the $+x$ direction, while the $\mathrm{q}$ and $\bar{\mathrm{q}}$ move in opposite directions along the *z* axis

Although the motion properties of the massless and massive cases hold in every longitudinal boosted frame, the effects of boosts are simpler to address for massless quarks. A useful trick is to replace the effect of the quark mass by an extra string piece of length $(E_{\mathrm{c}}(t) - p_{z,\mathrm{c}}(t))/\kappa$ at each endpoint. Its length is $m_{\mathrm{q}}/\kappa$ at the turning point, see Fig. 5b, where the massive motion is illustrated by the hyperbolae, whose asymptotes are the straight lines of the massless case. Thereby time $t = 0$ is also offset to account for the reduced oscillation time. The extra string piece is purely fictitious and does not break during the fragmentation process. The physical region, between the hyperbolae, is highlighted in grey in Fig. 5b. Given that the hadron created from the endpoint is always heavier than the endpoint quark, all the hadrons are automatically created inside the physical region.

The four-momenta of the massless reference four-vectors have to be linear combinations of the massive quark four-momenta for Lorentz covariance reasons. If $p_{\mathrm{q}}$ and $p_{\bar{\mathrm{q}}}$ are the four-momenta of the massive quarks, while $p_{0\mathrm{q}}$ and $p_{0\bar{\mathrm{q}}}$ are the massless four-momenta, the relation between them becomes

18 $$\begin{aligned} p_{0\mathrm{q}}= & {} (1+k_1) p_{\mathrm{q}} -k_2p_{\bar{\mathrm{q}}} ,\nonumber \\ p_{0\bar{\mathrm{q}}}= & {} (1+k_2) p_{\bar{\mathrm{q}}} - k_1p_{\mathrm{q}} , \end{aligned}$$

where the $k_1$ and $k_2$ values are fixed by $p_{0\mathrm{q}}^2 = p_{0\bar{\mathrm{q}}}^2 = 0$ [27].

### Multiparton systems

Next, more complicated string topologies need to be considered. An example is the $\mathrm{Z}^{0}$ decay into a pair of massless quarks, either of which can emit a gluon:

$$\begin{aligned} \mathrm{Z}^{0} \rightarrow \mathrm{q}\bar{\mathrm{q}}\rightarrow \mathrm{q}\mathrm{g}\bar{\mathrm{q}}. \end{aligned}$$

Both such radiation and the hadronization can occur over widely varying time scales in high-energy events, but in a local context the radiation takes place at time scales shorter than those of the hadronization itself. As a reasonable first approximation all three partons can thus be assumed created at the origin.

In the Lund model the colour flow is based on the limit of infinitely many colours [33]. Then there is one string piece from the $\mathrm{q}$ to the $\mathrm{g}$ and another from the $\mathrm{g}$ to the $\bar{\mathrm{q}}$, and the two do not interfere. The gluon thus can be viewed as a kink on a single string stretched from the $\mathrm{q}$ to the $\bar{\mathrm{q}}$.

The motion of the $\mathrm{q}\mathrm{g}\bar{\mathrm{q}}$ string system can conveniently be studied in a Lorentz frame where the $\mathrm{q}$ moves in the $+z$ direction, $\mathrm{g}$ in the $+x$ direction and $\bar{\mathrm{q}}$ in the $-z$ direction. Two string pieces are present initially, as illustrated in view 1 of Fig. 6. Each string piece defines a separate string region, which behaves similarly to the string piece of a $\mathrm{q}\bar{\mathrm{q}}$ system, except that it is now transversely boosted. The region formed by the $\mathrm{q}\mathrm{g}$ string evolves with time as

19 $$\begin{aligned} p_{\mathrm{q}}(t)= & {} (E_{\mathrm{q}}(0) - \kappa t) (1; 0, 0, 1) , \nonumber \\ p_{g}(t)= & {} (E_{\mathrm{g}}(0) - 2\kappa t) (1; 1, 0, 0) ,\nonumber \\ p_{\mathrm {string}}(t)= & {} \kappa t (2; 1, 0, 1) , \end{aligned}$$

and correspondingly for $\bar{\mathrm{q}}\mathrm{g}$, but with $z \rightarrow -z$. Note the factor of two in the gluon four-momentum, which comes from the loss of energy–momentum to both string pieces attached to it. Unlike the simple $\mathrm{q}\bar{\mathrm{q}}$ system, the two string pieces are not at rest, but move in the transverse direction: the $\mathrm{q}\mathrm{g}$ string piece has a velocity vector $v_x = v_z = 1/2$, while for the $\mathrm{g}\bar{\mathrm{q}}$ piece $v_x = -v_z = 1/2$. The energy per unit string length is higher than for a string at transverse rest, but the lower string length drawn out per unit time exactly compensates, such that the force acting on the endpoints is of the same magnitude [27].

After time $t = E_{\mathrm{g}}(0)/2 \kappa$ the gluon has lost all its energy and a new string piece is created by the inflowing momentum from the $\mathrm{q}$ and $\bar{\mathrm{q}}$, and is hence denoted as the $\mathrm{q}\bar{\mathrm{q}}$ region, see view 2. Later the $\mathrm{q}$ has also lost all its energy and starts to move in the $+x$ axis as it absorbs $\mathrm{g}$ four-momentum. The $\mathrm{q}$ eventually gains and re-emits half of the gluon energy, views 3 and 4. Subsequently it absorbs original $\bar{\mathrm{q}}$ four-momentum and moves in the $-z$ direction, views 5 and 6. A similar process occurs for $\bar{\mathrm{q}}$. As shown in view 7, the gluon will eventually reappear, and in view 8 the sequence starts to repeat, only with the momenta of $\mathrm{q}$ and $\bar{\mathrm{q}}$ swapped.

**Fig. 7:**
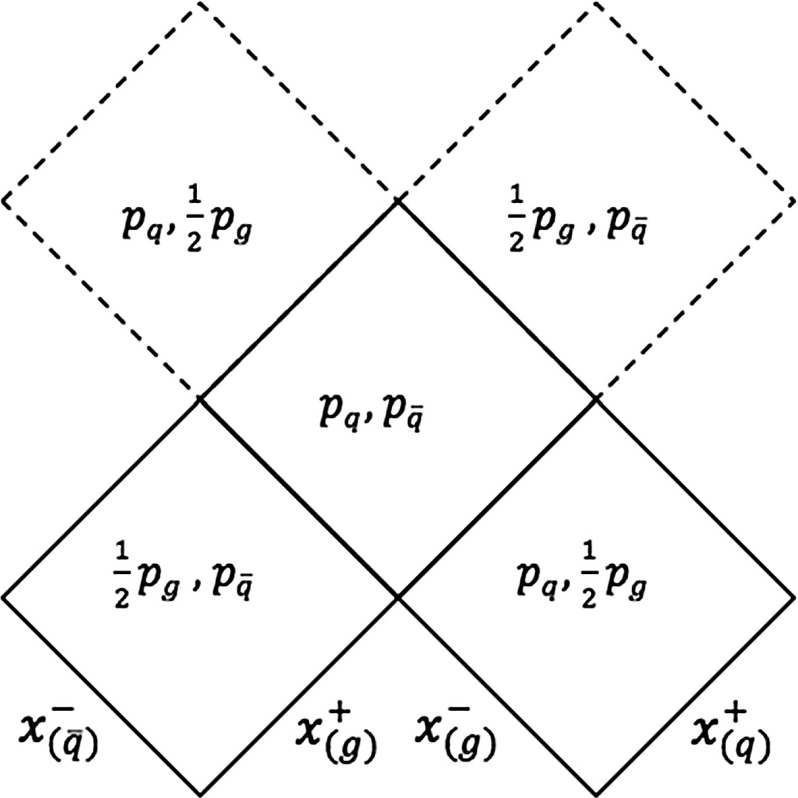
The parameter plane picture for the $\mathrm{q}\mathrm{g}\bar{\mathrm{q}}$ system. The dash lines indicate the turnover regions, normally neglected

Although Fig. 6 is useful to visualize the time evolution of the system, the parameter plane picture is most convenient when addressing the kinematics [27]. This is a diagram that displays the different string regions in terms of the light-cone four-vectors defining each region, i.e. $p^+_{\mathrm{q}} = p_{\mathrm{q}}$, $p^-_{\bar{\mathrm{q}}} = p_{\bar{\mathrm{q}}}$ and $p^+_{\mathrm{g}} = p^-_{\mathrm{g}} = p_{\mathrm{g}}/2$ in the $\mathrm{q}\mathrm{g}\bar{\mathrm{q}}$ case, whose parameter plane is displayed in Fig. 7. The low regions represent the states in which none of the partons have lost their energy, corresponding to the two string regions in view 1 of Fig. 6, the $\mathrm{q}\mathrm{g}$ and the $\mathrm{g}\bar{\mathrm{q}}$ string pieces. The intermediate region corresponds to the new string piece created from the $\mathrm{q}$ and $\bar{\mathrm{q}}$ momenta once the gluon has lost all its energy. Finally, the upper regions are related to the two string pieces formed when $\mathrm{g}$ re-appears. Although the complete parameter plane picture (for half a period) is the one shown in Fig. 7, the dashed upper regions are normally neglected, since the system is assumed to fragment before then. This reasonable assumption avoids a large number of complications for handling fragmentation in these regions. The three remaining regions are then formed by the combination of one $+$ component and one − one, where ± no longer relates to motion along the $\pm z$ axis, but more generically denotes the reference vector directed towards ($+$) or away from (−) the $\mathrm{q}$ end of the system.

From the parameter plane picture, the equations defining the hadron properties and the fragmentation process of Sect. 2.2 can be easily generalized. For the $\mathrm{q}\mathrm{g}\bar{\mathrm{q}}$ system, the hadron momentum can generically be written as,

20 $$\begin{aligned} p_h= & {} x^+_{\mathrm{q}} p^+_{\mathrm{q}} + x^-_{\mathrm{g}} p^-_{\mathrm{g}} + x^+_{\mathrm{g}} p^+_{\mathrm{g}} + x^-_{\bar{\mathrm{q}}} p^-_{\bar{\mathrm{q}}} , \nonumber \\= & {} x^+_{\mathrm{q}} p_{\mathrm{q}} + \frac{1}{2} \left( x^+_{\mathrm{g}}+ x^-_{\mathrm{g}}\right) p_{\mathrm{g}} + x^-_{\bar{\mathrm{q}}}p_{\bar{\mathrm{q}}} . \end{aligned}$$

The hadron mass enters via the constraint $p_h^2 = m_h^2$. The other Lorentz invariant variable $\varGamma$ is obtained from the $\hat{x}^{\pm }$ fractions defined in Sect. 2.2.2 as,

21 $$\begin{aligned} \varGamma = \left( \hat{x}^+_{\mathrm{q}} p^+_{\mathrm{q}} + \hat{x}^+_{\mathrm{g}} p^+_{\mathrm{g}} + \hat{x}^-_{\mathrm{g}} p^-_{\mathrm{g}} + \hat{x}^-_{\bar{\mathrm{q}}} p^-_{\bar{\mathrm{q}}}\right) ^2 . \end{aligned}$$

The level lines of constant $m_h$ and constant $\varGamma$ again give hyperbolae inside each string region, where physically allowed, which connect at the borderline between regions. As before there will be (at most) one allowed solution to a given ($m_h, \varGamma )$ pair, which can be found by starting in the current region and, if not found there, step by step move on to other possible regions. There are a number of complications that have to be overcome to do this [27].

**Fig. 8:**
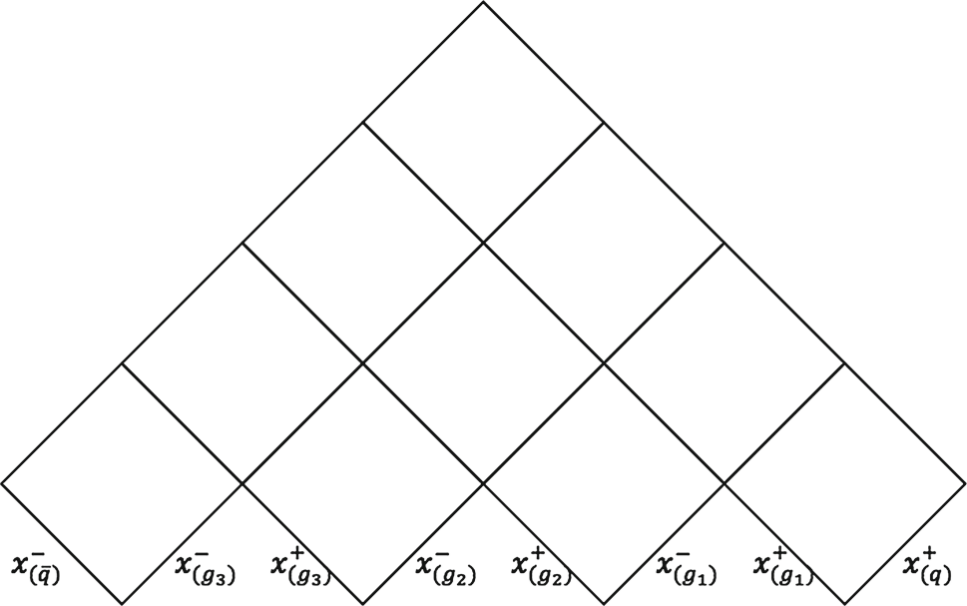
The parameter plane picture for a multiparton system composed by five partons

The parameter-plane picture can be extended to a multiparton system, resulting in the most convenient approach to study the kinematics of any multiparton system. As an example, the parameter plane for a system consisting of three gluons, one quark and one antiquark is depicted in Fig. 8, where the turnover regions have been ignored. In such a system, there are four low regions, or initial regions, and six intermediate regions. The number of initial and intermediate regions can be generalized for any multiparton system formed by *n* partons, out of which $n-2$ are gluons, as $n-1$ initial regions and $(n-1) (n-2)/2$ intermediate regions. The expression for the hadron four-momentum can also be generalized to an *n*-parton system by accounting for the momenta taken from each parton as

22 $$\begin{aligned} p_h = x_{\mathrm{q}}^+ p_{\mathrm{q}}^+ + x_{\bar{\mathrm{q}}}^- p_{\bar{\mathrm{q}}}^- + \sum _{i=1}^{n-2} \left( x_{g_i}^+ p^+_{g_i} + x_{g_i}^- p^-_{g_i}\right) , \end{aligned}$$

where usually most of the $x^{\pm }$ vanish. Apart from these aspects, the rest of the properties are similarly determined as in previous cases.

### Fragmentation implementation summary

The fragmentation process in Pythia is based on the four-momenta of the partons created in the (semi)perturbative stages of the collision process, plus the partons in the beam remnants [34]. By the colour-connection between those partons, an LHC event is likely to contain several $\mathrm{q}\mathrm{g}_1\mathrm{g}_2\ldots \mathrm{g}_{n-2}\bar{\mathrm{q}}$ systems, that can be handled separately.

The production of each new hadron begins with the selection at random of whether to split it off from the $\mathrm{q}$ end or from the $\bar{\mathrm{q}}$ one of the system. The flavour of a new $\mathrm{q}\bar{\mathrm{q}}$ break of the string (where $\mathrm{q}$ may also represent an antidiquark), leads to the formation of a new hadron, as already described. Its mass is selected, according to a Breit–Wigner for short-lived particles with a non-negligible width. The transverse momentum is obtained as the vector sum of those of the hadron constituents, assuming that the old and new breakup vertices are in the same region. Then the longitudinal momentum fraction *z* is picked according to the probability distribution in Eq. (12), with the difference that the hadron mass has to be replaced by the transverse ditto, $m_{h} \rightarrow m_{\perp h}$. In a simple $\mathrm{q}\bar{\mathrm{q}}$ system, the new breakup vertex is easily obtained from the $(m_{\perp h},z)$ pair. Else the $\varGamma _i$ value of the new breakup is calculated using Eq. (13), again with $m_{h} \rightarrow m_{\perp h}$, and a solution is sought to the $(m_{\perp h}, \varGamma _i)$ pair of equations. Vertex *i* may end up in the same string region as $i - 1$, or involve a search in other regions. Among technical complications of this search is that the transverse directions are local to each string region, which leads to discontinuities in the hyperbolae of constant $m_{\perp h}$ at the borders between string regions, that would not be there for $p_{\perp }= 0$.

The random steps from both string ends continue until the remaining invariant mass of the system is deemed so small that only two final hadrons should be produced. Details on this final step can be found in “Appendix A”, along with the challenges encountered when implementing the space–time picture and the methods applied to solve them. Had the fragmentation always proceeded from the $\mathrm{q}$ end, say, the final step would always have been at the $\bar{\mathrm{q}}$ end, with the minor blemishes of this step concentrated there. Now these are instead smeared out over the whole event.

## The space–time description

So far, the fragmentation process in Pythia was developed in terms of the energy–momentum fractions $x^{\pm }$ and $z^{\pm }$ of breakup vertices and hadrons, presented in Sect. 2.2.2. Therefore, the location of the breakup vertices is only specified in the energy–momentum picture. In order to study the density of hadron production, this information should first be translated to the space–time one, which will be done in this section.

### The two-parton system

To begin, consider a breakup point *i* in a simple $\mathrm{q}\bar{\mathrm{q}}$ system. Its location with respect to the origin of the energy–momentum picture, where $\mathrm{q}$ and $\bar{\mathrm{q}}$ have been created, is given by the $\hat{x}^{\pm }$ fractions. Then, considering $p^+$ to be the $\mathrm{q}$ four-momentum and $p^-$ the $\bar{\mathrm{q}}$ four-momentum, the location of breakup *i* in the energy–momentum picture is defined as $\hat{x}_i^+ p^+ + \hat{x}_i^- p^-$. Recalling the linear relation between space–time and energy–momentum, Eq. (3), the space–time location of breakup point *i* thereby is defined as

23 $$\begin{aligned} v_i = \frac{\hat{x}_i^+ p^+ + \hat{x}_i^- p^-}{\kappa } . \end{aligned}$$

Note that the $\mathrm{q}_i$ and $\bar{\mathrm{q}}_i$ generated by the string break are considered to be created in the same space–time vertex, even when quark masses and transverse momenta are included, such that $\mathrm{q}$ and $\bar{\mathrm{q}}$ have to tunnel some distance apart before becoming on-shell. Such effective vertices in practice is the best one can do.

**Fig. 9:**
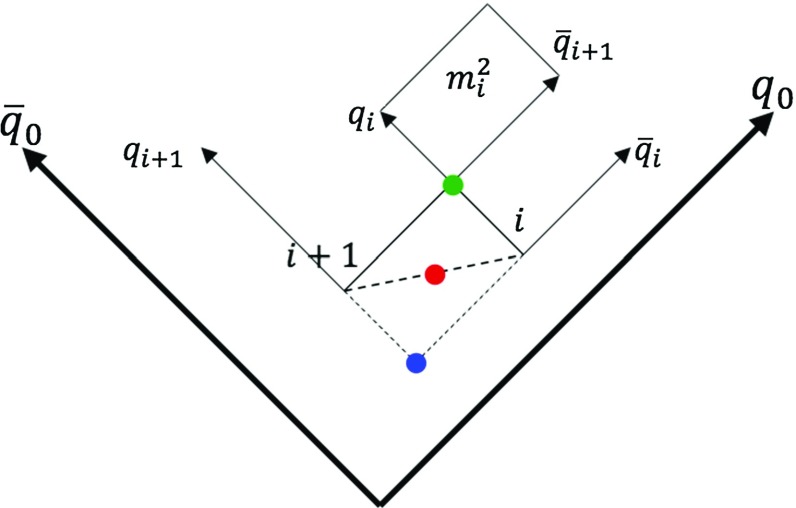
Hadron formation in a $\mathrm{q}\bar{\mathrm{q}}$ system. The blue, red and green dots represent the “early”, “middle” and “late” definitions of hadron production points, respectively

Since hadrons are formed by two adjacent string breaks, the hadron production point should be related to these two. But the definition cannot be unique, since hadrons are composite and extended particles. For that reason, we propose three alternative definitions, illustrated in Fig. 9. Two breakup points, *i* and $i+1$, with space–time coordinates $v_i$ and $v_{i+1}$, together define the $\mathrm{q}_i\bar{\mathrm{q}}_{i+1}$ subsystem that forms hadron *i*. One obvious choice is then to define the hadron production point as the average of the two,

24 $$\begin{aligned} v^h_i = \frac{v_i + v_{i+1}}{2} , \end{aligned}$$

red dot in the figure. Alternatively to this “middle” definition, the “late” hadron production point is where the two partons forming the hadron cross for the first time, green dot. Taking into account the hadron four-momentum $p_h$, the “late” hadron production point is offset from the “middle” definition as

25 $$\begin{aligned} v^h_{l,i} = \frac{v_i + v_{i+1}}{2} + \frac{p_h}{2\kappa } . \end{aligned}$$

Finally, an “early” definition, blue dot, is given by

26 $$\begin{aligned} v^h_{e,i} = \frac{v_i + v_{i+1}}{2} - \frac{p_h}{2\kappa }, \end{aligned}$$

which is where the backwards light cones of the $\bar{\mathrm{q}}_i$ and $\mathrm{q}_{i+1}$ vertices cross, just like the “late” definition is where the forwards light cones cross. In a causal world, this would be the latest time for information to be sent out that can correlate the breakup vertices to give the correct hadron mass. Note that the two endpoint hadrons are situated on the light cone with this “early” definition. The different results obtained with the three alternative definitions can be used as a measure of uncertainty, see Sect. 4.1. If not stated otherwise, the choice in this article is the “middle” definition of Eq. (24).

### More complex topologies

**Fig. 10:**
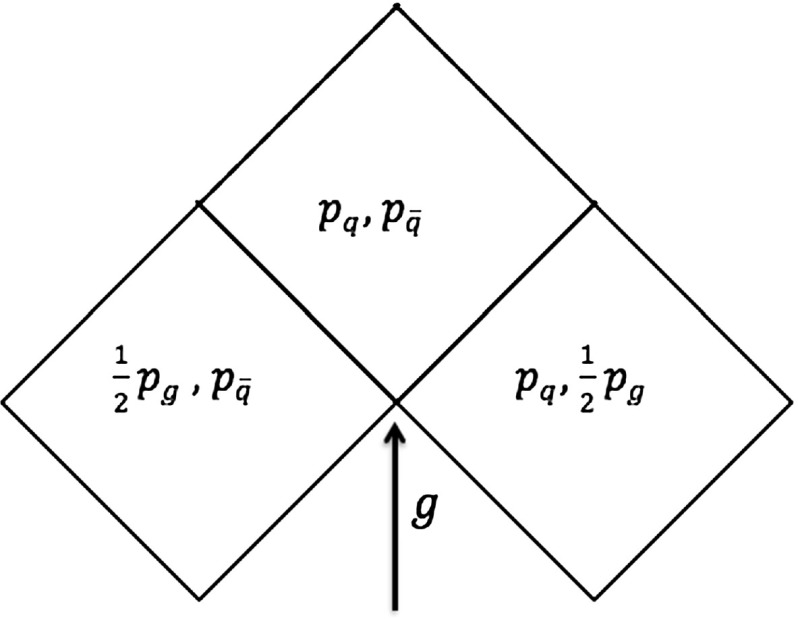
Parameter plane for a $\mathrm{q}\mathrm{g}\bar{\mathrm{q}}$ system

Multiparton systems are more complicated to address than a single $\mathrm{q}\bar{\mathrm{q}}$ string, as already demonstrated for the energy–momentum picture. Their complexity also affects the space–time implementation, which has to be extended to include several string regions. Initially consider a $\mathrm{q}\mathrm{g}\bar{\mathrm{q}}$ system formed by massless partons, with a parameter plane as in Fig. 10, ignoring the turnover regions. Since each string region separately behaves like a simple $\mathrm{q}\bar{\mathrm{q}}$ system, Eq. (23) can be used. Nevertheless, the intermediate region is formed after the gluon has lost all of its energy, at a different location in space–time than the initial regions, so an offset has to be taken into account for it. From the linear relation between space–time and energy–momentum, the space–time offset for this region can be calculated as $v_{\mathrm {reg}} = p_{\mathrm{g}}/2\kappa$, where $p_{\mathrm{g}}$ is the four-momentum of the gluon. The factor of 1 / 2 accounts for the fact that a gluon loses four-momentum twice as fast as a $\mathrm{q}$ or $\bar{\mathrm{q}}$, since it transfers four-momentum to two string pieces. Thus, the space–time location of a breakup located in the intermediate region is given by

27 $$\begin{aligned} v_i = \frac{\hat{x}_i^+ p^+ + \hat{x}_i^- p^-}{\kappa } + \frac{p_{\mathrm{g}}}{2\kappa } . \end{aligned}$$

**Fig. 11:**
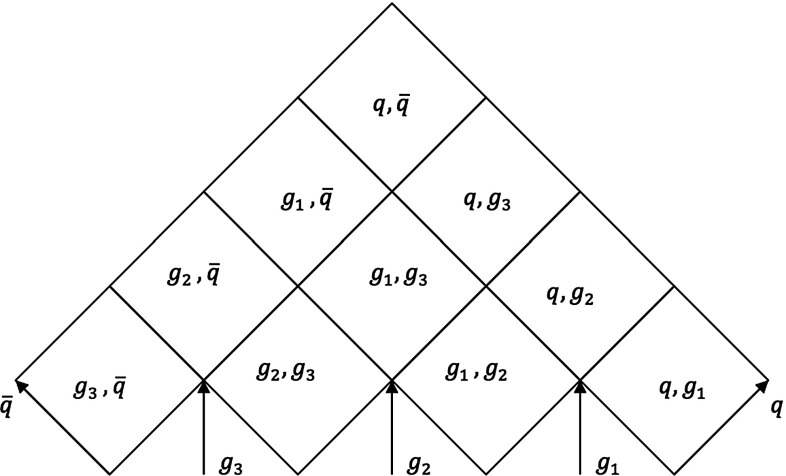
Parameter plane for a five parton system

If the system is composed of more than one gluon, also more than one intermediate region has to be taken into account, as illustrated in Fig. 11. In such cases, more gluons have to be included when determining the space–time offset of some intermediate regions, such as the $\mathrm{q}\mathrm{g}_3$ one. This region is created when both $\mathrm{g}_1$ and $\mathrm{g}_2$ have lost their energies, giving an offset $v_{\mathrm {reg}} = (p_{g_1}+p_{g_2}) /2\kappa$.

A general expression for the space–time offset of any intermediate region in any multiparton system can be easily defined, if all partons are numbered consecutively, starting from the $\mathrm{q}$ end, and region labels *jk* are for ones containing four-momenta from partons *j* and $k, k\ge j$. The *jk* region offset is found to be

28 $$\begin{aligned} v_{jk} =\sum _{m = j + 1}^{k - 1} \frac{p_m}{2\kappa } , \end{aligned}$$

where $p_m$ is the four-momentum of parton *m*, and for a breakup vertex in this region it thus holds that,

29 $$\begin{aligned} v_i = \frac{\hat{x}_i^+ p^+_j + \hat{x}_i^-p^-_k}{\kappa } + \sum _{m = j + 1}^{k - 1}\frac{p_m}{2\kappa }. \end{aligned}$$

While seemingly simple enough, there are a number of significant challenges to a robust implementation in a multiparton configuration, in part paralleling similar problems in the energy–momentum picture [27], in part going further. There are two main problems: the determination of the space–time location of the final breakup in the system, and the non-physical values of the $\hat{x}^{\pm }$ fractions that can arise when fragmentation moves to a new region. Those issues are further explained in “Appendices A and B”, respectively, along with the solutions found to properly implement the space–time picture.

**Fig. 12:**
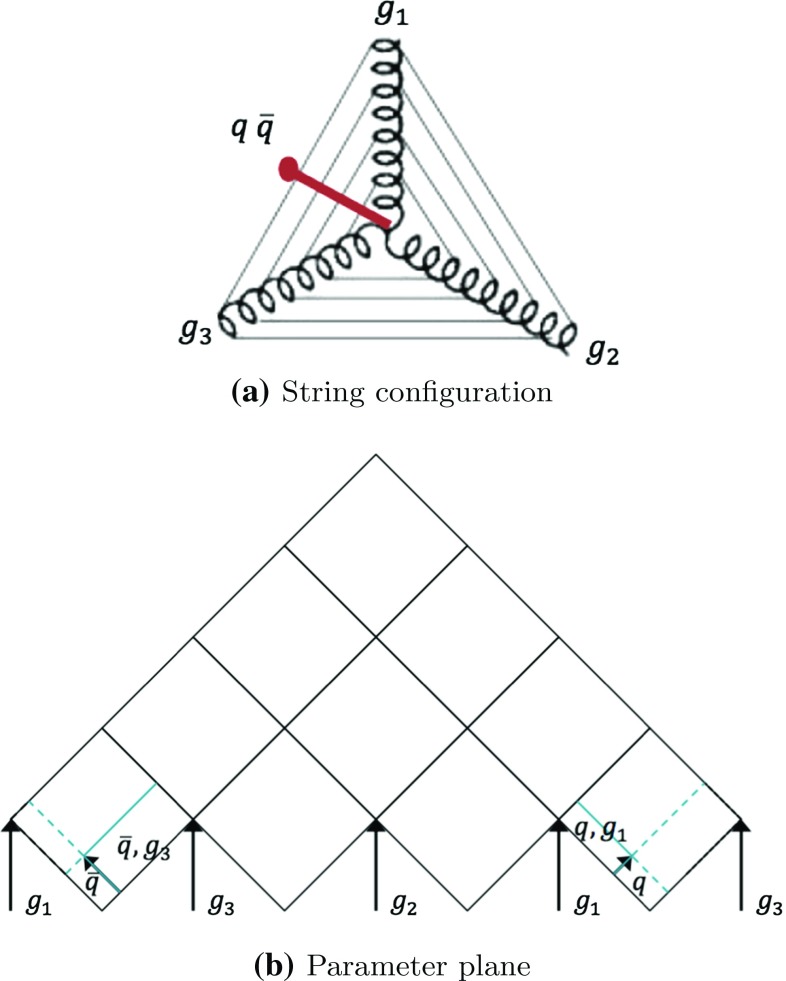
The string configuration and the corresponding parameter plane for a three gluon-loop topology. In the two initial endpoint regions the full lines indicate the “active area”, and the dashed ones the complementary excluded one

### Gluon loops

So far, gluons have only appeared in open strings between a $\mathrm{q}$ and a $\bar{\mathrm{q}}$ end, but it is also possible to have closed gluon loops, as exemplified in Fig. 12a for a $\mathrm{g}\mathrm{g}\mathrm{g}$ system. In order to reduce the problem to a familiar one, an initial $\mathrm{q}\bar{\mathrm{q}}$ is generated by string breaking in one of the string regions. This break should be representative of what ordinary fragmentation is expected to give. Thus the region is chosen at random, but with a bias towards ones with larger masses, where more ordinary string breaks are to be expected. Inside that region, the $\varGamma$ value of the vertex is chosen according to Eq. (14), and a further random choice gives the longitudinal location of the breakup. Having taken this step, the *n*-gluon-loop topology is effectively mapped onto an $(n+1)$-parton open string with $\mathrm{q}$ and $\bar{\mathrm{q}}$ as endpoints. The key difference is that, unlike open strings considered so far, $\varGamma _{\mathrm{q}} = \varGamma _{\bar{\mathrm{q}}} \ne 0$.

As an example, the parameter plane for a gluon-loop consisting of three gluons is displayed in Fig. 12b. In this case, the string between $\mathrm{g}_1$ and $\mathrm{g}_3$ has broken into two string pieces, generating two new string regions, $\mathrm{g}_1\mathrm{q}$ and $\mathrm{g}_3\bar{\mathrm{q}}$. Although the full $\mathrm{g}_1\mathrm{g}_3$ region is duplicated in the parameter plane, in the right endpoint region only the “active area” between $\mathrm{q}$ and $\mathrm{g}_1$ is open to fragmentation, while the left endpoint region only uses the complementary area between $\mathrm{g}_3$ and $\bar{\mathrm{q}}$. Apart from that, the fragmentation process can now play out in the same way as for an open string, with the same rules for the space–time locations of the breakups. Note that the $\mathrm{q}$ and $\bar{\mathrm{q}}$ “endpoints” correctly will be assigned the same creation vertex in this procedure.

### Smearing in transverse space

Strings can be viewed as the center of cylindrical tubelike regions of directed colour flow. So far we have assigned production vertices as if they all were in the very center of the string. A more realistic picture is to introduce some transverse smearing. For simplicity this is done according to a two-dimensional Gaussian

30 $$\begin{aligned} f (x,y) \propto \exp \left( -\frac{x^2+y^2}{2\sigma ^2} \right) , \end{aligned}$$

where *x* and *y* are transverse spatial coordinates and $\sigma$ is the width of the distribution.

The width of the string should be of typical hadronic scales, but related to confinement in two dimensions rather than three. Taking the proton radius $r_{\mathrm{p}}\approx 0.87$ fm [35] as starting point, the default $\sigma = r_{\mathrm{p}}/\sqrt{3}$ then gives a,

31 $$\begin{aligned} r_{\perp ,\mathrm{p}}^2 = \langle x^2+y^2 \rangle = 2 \sigma ^2 = \frac{2}{3} r_{\mathrm{p}}^2. \end{aligned}$$

The smearing in transverse space might generate unwanted situations, such as negative values for the $\varGamma$ parameter of the breakup points. Since the space–time location is first obtained from the fragmentation picture in the longitudinal direction, the squared invariant time should not change when introducing smearing. Therefore, the time coordinate is adjusted after including the smearing in transverse space, in order to retain the $\varGamma$ value determined by the longitudinal scheme. Alternative procedures could be envisioned, in particular when the collision process itself does not happen in the origin, but for now this smearing possibility is good enough to indicate trends.

### Massive quark implementation

As illustrated in Sect. 2.2.5, the origin of the massive and massless oscillations are displaced for technical reasons; correspondingly the initial point of the massive oscillation is offset from the origin of the space–time coordinate system. Since the fragmentation process is performed in the massless system, the space–time locations of the breakups have to be adjusted.

**Fig. 13:**
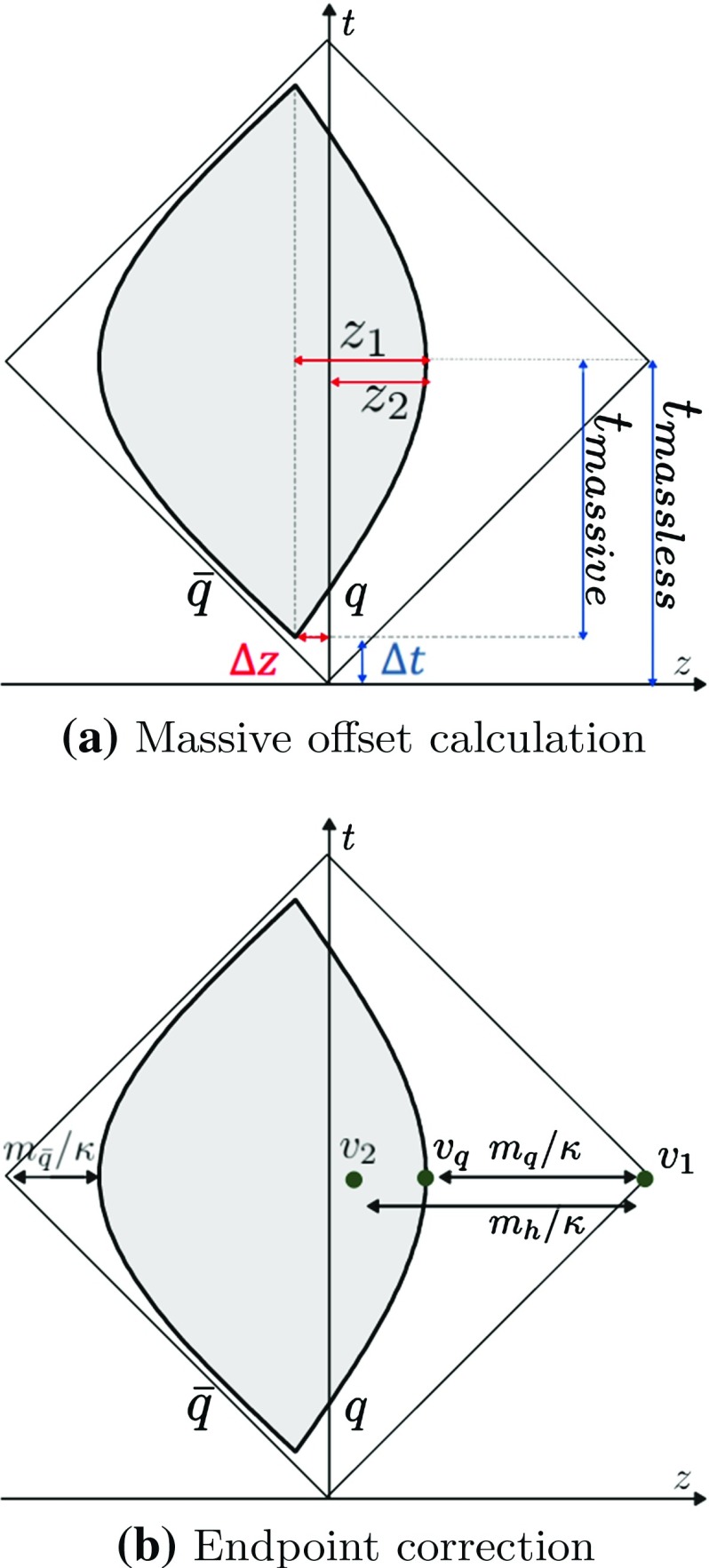
Massive $\mathrm{q}\bar{\mathrm{q}}$ system and equivalent massless system. The grey area corresponds to the physical region

To determine the offset, consider the $\mathrm{q}\bar{\mathrm{q}}$ system in Fig. 13a, studied in the CM frame, with $\mathrm{q}/\bar{\mathrm{q}}$ moving in the $\pm z$ directions. In this case the $\mathrm{q}$ and $\bar{\mathrm{q}}$ masses are different, with $m_{\mathrm{q}} > m_{\bar{\mathrm{q}}}$. At time $t=0$ we have $p_0 = p_{z,\mathrm{q}} = -p_{z,\bar{\mathrm{q}}}$ and $E_{\mathrm{cm}}= E_{\mathrm{q}} + E_{\bar{\mathrm{q}}}$, with $p_0$, $E_{\mathrm{q}}$ and $E_{\bar{\mathrm{q}}}$ given by standard two-body decay kinematics. The massive oscillation in Fig. 13a is offset both in time and *z*-component of space, represented as $\varDelta t$ and $\varDelta z$. The former can be determined from the difference between the time coordinates at which the massless and massive quarks lose their three–momenta, $t_{\mathrm {massless}}$ and $t_{\mathrm {massive}}$ in Fig. 13a, i.e. 

32 $$\begin{aligned} \varDelta t = t_{\mathrm {massless}} - t_{\mathrm {massive}} = \frac{E_{\mathrm{cm}}}{2\kappa } - \frac{p_0}{\kappa } = \frac{E_{\mathrm{cm}}- 2p_0}{2\kappa }.\nonumber \\ \end{aligned}$$

The process to define the space offset is slightly different. In Fig. 13a, the distance of the massive $\mathrm{q}$ endpoint to the centre of the massive oscillation is denoted $z_1$, while $z_2$ is the distance of the massive $\mathrm{q}$ endpoint to the centre of the massless system. The equation of motion then gives,

33 $$\begin{aligned} \varDelta z= & {} z_1 - z_2 = \frac{1}{\kappa } \left( \frac{E_{\mathrm{cm}}}{2} - m_{\mathrm{q}} \right) - \frac{1}{\kappa } (E_{\mathrm{q}}-m_{\mathrm{q}})\nonumber \\= & {} \frac{E_{\bar{\mathrm{q}}} - E_{\mathrm{q}}}{2\kappa } . \end{aligned}$$

The time and space offsets can be combined as,

34 $$\begin{aligned} v_{\mathrm {offset}} = \frac{1}{\kappa E_{\mathrm{cm}}} \left( (E_{\bar{\mathrm{q}}} - p_0) p_{0\mathrm{q}} + (E_{\mathrm{q}} - p_0) p_{0\bar{\mathrm{q}}} \right) , \end{aligned}$$

where $p_{0\mathrm{q}}$ and $p_{0\bar{\mathrm{q}}}$ are the four-vectors of the equivalent massless system, cf. Eq. (18). Hence, for each vertex in a region formed by at least one massive quark, the space–time location is defined as usual from the massless system, $v_{\mathrm {massless}}$, and then corrected to,

35 $$\begin{aligned} v_{\mathrm {correct}} = v_{\mathrm {massless}} - v_{\mathrm {offset}} . \end{aligned}$$

For more complex topologies, such as multiparton systems consisting of a massive $\mathrm{q}$ and/or $\bar{\mathrm{q}}$ and several gluons, the effect of the massive $\mathrm{q}$ or $\bar{\mathrm{q}}$ is only non-negligible in the lowest respective endpoint region. Therefore, the massive correction is only performed in those regions.

Also the space–time location of the massive endpoint quark “vertex” has to be offset, away from what it would have been for a massless quark. This is exemplified in Fig. 13b, for the same massive $\mathrm{q}\bar{\mathrm{q}}$ system as before. The three vertices $v_1$, $v_{\mathrm{q}}$ and $v_2$ correspond to the space–time location of the massless endpoint, the massive turning point and the closest breakup to the massless endpoint, respectively. The system can be studied in a Lorentz frame where the three vertices are simultaneous, $v_{1,t} = v_{2,t} = v_{\mathrm{q},t}$. Then, linearity between energy–momentum and space–time gives,

36 $$\begin{aligned}&v_{1,z} - v_{2,z} = \frac{m_h}{\kappa } ,\nonumber \\&v_{1,z} - v_{\mathrm{q},z} = \frac{m_{\mathrm{q}}}{\kappa } , \end{aligned}$$

where $m_{\mathrm{q}}$ is the mass of the heavy quark and $m_h$ the mass of the hadron formed from the vertices $v_1$ and $v_2$. From this $v_{z,\mathrm{q}}$ can be extracted. Recast in Lorentz-invariant four-vector notation, this gives,

37 $$\begin{aligned} v_{\mathrm{q}} = v_1 + \frac{m_{\mathrm{q}}}{m_h} (v_2 - v_1). \end{aligned}$$

Note that, after the correct endpoint location has been determined, the offset correction of Eq. (35) has to be included. If a system is formed by a massless $\mathrm{q}$ and a massive $\bar{\mathrm{q}}$, say, Eq. (37) has to be applied only to the massive $\bar{\mathrm{q}}$, while the offset in Eq. (35) has to be used both for $\mathrm{q}$ and $\bar{\mathrm{q}}$.

A final feature is that the oscillation period for a hadron composed of massive quarks is shorter than a same-mass one with massless quarks. This discrepancy only affects the estimation of the “late”, $v_{l}^h$, and “early”, $v_{e}^h$, definitions of hadron production points. The expression in Eqs. (25) and (26) now become,

38 $$\begin{aligned} v_{l/e}^h = \frac{v_i + v_{i+1}}{2} \pm \alpha _{\mathrm {red}} \, \frac{p_h}{2\kappa } , \end{aligned}$$

where $\alpha _{\mathrm {red}}$ accounts for the reduced oscillation period. This parameter is determined in the hadron rest frame by the absolute three-momentum of the quarks forming the hadron

39 $$\begin{aligned} \alpha _{\mathrm {red}} = \frac{p_0}{m_h} = \frac{\sqrt{ \left( m_h^2 - m_{\mathrm{q}}^2 - m_{\bar{\mathrm{q}}}^2\right) ^2 - 4m_{\mathrm{q}}^2 m_{\bar{\mathrm{q}}}^2}}{m_h^2} , \end{aligned}$$

where $m_h$ is the mass of the hadron and $m_{\mathrm{q}}$ and $m_{\bar{\mathrm{q}}}$ the masses of the constituent $\mathrm{q}$ and $\bar{\mathrm{q}}$, respectively. Needless to say, these semiclassical estimates of oscillation periods cannot be taken too literally. It could be argued that all hadrons, light as heavy, have hadronic sizes of order 1 fm, and should have essentially common oscillation periods related to that. That would give us problems notably for pions, however, which are abnormally light in relation to their size.

### Other implementation details

Up until now, only open $\mathrm{q}\mathrm{g}_1\mathrm{g}_2\ldots \mathrm{g}_{n-2}\bar{\mathrm{q}}$ and closed $\mathrm{g}_1\mathrm{g}_2\ldots \mathrm{g}_n$ strings have been considered. A third possibility is junction topologies, wherein three string pieces meet in a common vertex [36], and whereby the junction effectively carries the baryon number of the system. Such topologies can arise e.g. when the three valence quarks are all kicked out of an incoming proton, but there are also scenarios in which further junctions and antijunctions may be formed [37].

A junction system consists of three different “legs”, each stretched from an endpoint quark via a number of gluons in to the junction. In Pythia the fragmentation process is most conveniently defined in the rest frame of the junction. Here the total energy of each leg is determined, and the two legs with the lowest energies are fragmented from the respective $\mathrm{q}$ end inwards. The process stops when the next step would require more energy than left in the leg. Once the two initial legs have fragmented, the two leftover $\mathrm{q}$ from the respective last breaks are combined to create a diquark. Together with the third leg and its original endpoint $\mathrm{q}$, this diquark defines a final string system, which now fragments as a normal open string.

The assignment of space–time locations in junction topologies introduces no new principles, but requires some extra bookkeeping. The three junction legs are considered as three different systems, to be dealt with in the same order as they fragmented, starting from the leg with the lowest energy.

Low-invariant-mass systems hadronize about as high-mass ones, even if kinematics is more constrained. The exception is when the invariant mass of the system is so low that only one hadron can be formed. In such cases, the “early” hadron production point is at the origin of the $\mathrm{q}\bar{\mathrm{q}}$ system, i.e. $v_e^h= (0; 0, 0, 0)$. Note that smearing in transverse space will give rise to negative squared invariant times in such cases. This is not a problem if the reason is that the collision of two Lorentz-contracted proton “pancakes” naturally would lead to a spread of *x*, *y* coordinates of collisions at $t=0$. The “middle” and “late” definitions are calculated from the four-momentum $p_h$ of the hadron as $v^h = p_h/2\kappa$ and $v_l^h = p_h/\kappa$, respectively.

Finally, many of the hadrons produced during the string fragmentation are unstable and decay further, a process known as secondary particle production. In such cases the invariant lifetime is selected at random according to an exponential decay, $P(\tau ) \propto \exp (-\tau /\langle \tau \rangle )$, where $\langle \tau \rangle$ is the tabulated average lifetime [35]. For short-lived particles it is rather the width $\varGamma$ of the mass distribution that is known, and then one can use $\langle \tau \rangle = \hbar c/\varGamma$. Given a known hadron production vertex, the decay one becomes

40 $$\begin{aligned} v_{\mathrm {decay}} = v_{\mathrm {production}} + \tau \, \frac{p_h}{m_h} , \end{aligned}$$

for a hadron with four-momentum $p_h$ and mass $m_h$. This equation can be used recursively through decay chains, also e.g. for leptons.

Truly stable particles are only $\mathrm{e}^{\pm }$, $\mathrm{p}$, $\bar{\mathrm{p}}$, $\gamma$ and the neutrinos. Also some weakly decaying particles with long lifetimes are effectively treated as stable by default: $\mu ^{\pm }$, $\pi ^{\pm }$, $\mathrm{K}^{\pm }$, $\mathrm{K}_L^0$ and $\mathrm{n}/\bar{\mathrm{n}}$.

### A comparison of time scales

In this article we only address the space–time picture of hadronization. In the context of a hard collision process, say $\mathrm{q}\mathrm{g}\rightarrow \mathrm{q}\mathrm{g}$, also perturbative emission of further partons off the two scattered partons is extended in space and time. This is related to the regeneration time of the QCD field, mainly consisting of gluons, at typical time scales of order,

41 $$\begin{aligned} t_{\mathrm {regen}} \sim \frac{\hbar c \, E}{p_{\perp }^2} = \frac{\hbar c}{p_{\perp }} \, \frac{E}{p_{\perp }} \sim \tau _{\mathrm {regen}} \, \gamma \end{aligned}$$

for emitted partons of energy *E* and transverse momentum $p_{\perp }$ [38]. This expression is conveniently split into a “Heisenberg uncertainty” factor ($p_{\perp }$ is a measure of the off-shellness of intermediate propagators) and a “time dilation” factor, as indicated. Similar relations hold for emissions off the two incoming partons.

Typically, parton shower descriptions in event generators such as Pythia stop at scales of the order $\mathrm{p}_{\perp \mathrm {min}}\,=\,$0.5–1 GeV, mainly because $\alpha _{\mathrm{s}}$ becomes so big that perturbation theory cannot be trusted below that. (The current default value for Pythia final-state radiation is 0.5 GeV, but that is the $p_{\perp }$ for each daughter of a branching with respect to the mother direction, meaning a separation of 1 GeV between the two daughters. Eq. (41) should not be trusted up to factors of 2 anyway.) This corresponds to a $\tau _{\mathrm {regen}} \approx 0.25$ fm, say, to be compared with the average hadronization time $\langle \tau _{\mathrm {had}} \rangle \approx 1.3$ fm (see Sect. 4.2 below), i.e. about a factor five difference. To a good first approximation, the simulated perturbative activity can therefore be viewed as happening in a single point as far as the hadronization process is concerned. This is even more so for the hard perturbative activity that gives rise to separate jets, for which $p_{\perp }\gg 1$ GeV. The emissions that possibly are simulated below 1 GeV can only give small wrinkles on the strings stretched between the main partons.

The comparison of invariant time generalizes to hold everywhere in an event, since time dilation works the same way for showers and hadronization. That is, a perturbative splitting at high energy and low $p_{\perp }$ may occur at large time scales as measured in the rest frame of the event, when hadronization already started in the central region, but still well before it will begin in the part of the event that could be affected by the splitting.

At the end of the Pythia showers, the total number of partons in a typical LHC event is roughly half of the number of primary hadrons later produced. Given that the size, in each of three spatial dimensions, is only a fifth for the partonic system compared with the hadronic one, it might seem that the partonic density is much higher than the the hadronic one, and that partonic close-packing would be a more severe issue than hadronic ditto. Partons don’t have a well-defined size, however. A newly created parton could be assigned a vanishingly small size, and then the colour field surrounding it would expand with the speed of light. Thus the partonic size could be equated with the time since creation, multiplied by a standard time dilation factor.

At early times the partonic system of a collision therefore expands in size at about the same rate as the size of partons, and any net effect comes from the rise of the total number of partons as the cascade evolves from early times. Here the colour coherence phenomenon enters, however [38]. It is the obervation that the two daughters of a $\mathrm{q}\rightarrow \mathrm{q}\mathrm{g}$ or $\mathrm{g}\rightarrow \mathrm{g}\mathrm{g}$ branching share a newly-created colour-anticolour pair, that cannot contribute to the radiation until the partons are more separated than the wavelength of the further radiated partons. This gives a mechanism for close-packing avoidance, in event generators implemented in terms of angular or $p_{\perp }$ ordering of radiation.

Had the parton shower been allowed to evolve further than the current cutoff, the partonic multiplicity and the partonic overlap would have increased as the $\Lambda _{\mathrm {QCD}}$ scale of $\approx 0.3$ GeV is approached. By then the naive size of partons would be of the order of 0.7 fm, which is about the expected transverse size of strings, and soft partons emitted at this stage form part of the emergent strings. We do not know how to model these late stages of the cascade, but any effects coming from them are included in the tuned parameters of the string fragmentation framework.

The picture painted here is based on studying one partonic cascade. Since protons are composite object, however, several partonic subcollisions can occur when two protons collide – MPIs. One therefore also should worry about the overlap of cascades from different MPIs – partonic rescattering. In part this issue has been studied [39], and shown to give small effects. That study only included the effects of parton multiplication by initial-state radiation, as encoded in parton distribution functions, and thus did not address the effects of collisions between partons from two separate MPI subcollisions. In general, however, MPIs occur at different transverse locations when the two Lorentz-contracted protons collide, and the products move out in different rapidities and azimuthal directions. Also here it is therefore plausible with only minor overlap at early times and large perturbative scales. (In a relative sense; most MPIs do not have all that large $p_{\perp }$ values.) The overlap becomes relevant at later scales, where colour reconnection is the currently favoured mechanism for interactions between the emerging colour fields.

Another issue that we would like to comment on is the folklore that “fast particles are produced early”. This would seem to be in contradiction with the string picture, where hadronization begins with slow particles in the central region and then spreads outwards to faster particles at later times, roughly along a hyperbola of constant invariant time. But it is all a matter of what comparison one has in mind, and what production time definition is used [40]. Consider the “first” (“leading”) hadron, i.e. the one closest to the quark end of a $\mathrm{q}\bar{\mathrm{q}}$ string. For it $\varGamma _{i - 1} = \varGamma _0 = 0$, such that Eqs. (13) and (9) together give

42 $$\begin{aligned} \varGamma _1 = \frac{1 - z_1}{z_1} \, m_1^2 = (\kappa \tau _1)^2. \end{aligned}$$

The faster the hadron, the earlier the string break in invariant time: $\varGamma _1 \rightarrow 0$ for $z_1 \rightarrow 1$. Also the time in the string rest frame,

43 $$\begin{aligned} \kappa t_1 = E_{\mathrm{q}} (1 - z_1) + \frac{m_1^2}{4 E_{\mathrm{q}} z_1}, \end{aligned}$$

with $E_{\mathrm{q}}$ the quark energy, is decreasing for increasing $z_1$.

This reasoning generalizes: an event with few, fast particles can only be obtained when the $\varGamma$ values and the breakup times are small. Conversely, events with high multiplicities of lower-momentum hadrons require high $\varGamma$ values and late hadronization times. Whether early or late invariant times, however, the hadronization will still start in the middle and spread outwards.

## Hadron density studies

We now proceed to study the implications of the model presented so far. Toy studies are reported for a simple $\mathrm{q}\bar{\mathrm{q}}$ string, but most results are for $\mathrm{p}\mathrm{p}$ collisions at $\sqrt{s} = 13$ TeV, for inclusive inelastic nondiffractive events. Although the $\mathrm{p}\mathrm{p}$ modelling is not yet complete, enough is in place to perform some first semi-realistic studies that form the basis for future development. Notably we will estimate the hadronic density in a few different ways, as a means to highlight the close-packing of hadrons and the need to consider the consequences of that.

**Fig. 14:**
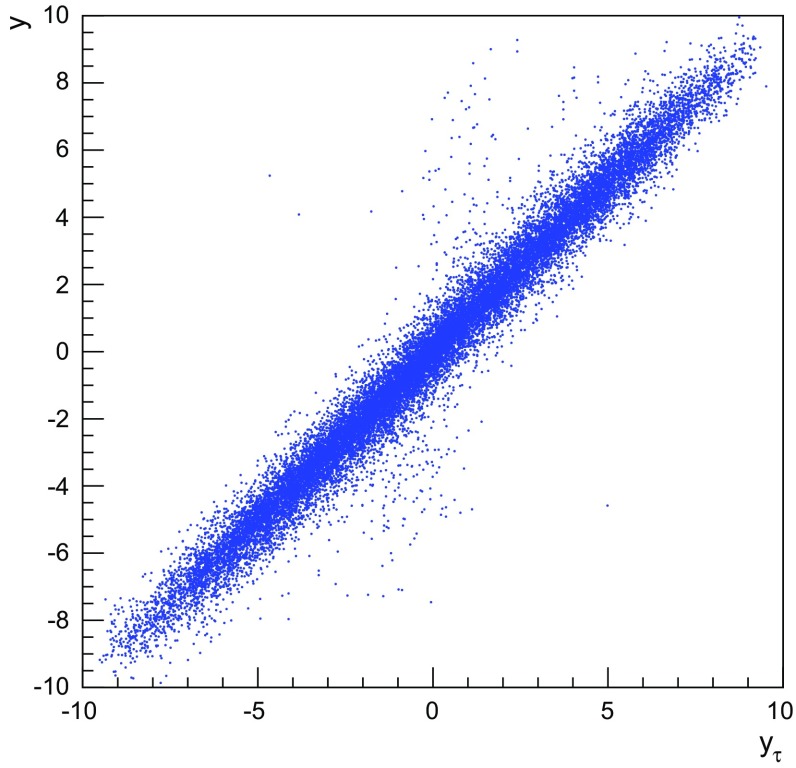
Correlation between rapidity, *y*, and the equivalent space–time rapidity, $y_{\tau }$, for all hadrons in 100 inelastic nondiffractive $\mathrm{p}\mathrm{p}$ events at $\sqrt{s}=13$ TeV

### Longitudinal and transverse distributions

Three definitions of hadron production points were presented in Sect. 3.1, to allow estimates of the uncertainty in the description. Here the three resulting longitudinal and transverse space–time distributions are compared. For the former $y_{\tau }$ is introduced as a space–time correspondent to ordinary rapidity *y*:

44 $$\begin{aligned} y = \frac{1}{2} \log \left( \frac{E + p_z}{E - p_z} \right) \longrightarrow y_{\tau } = \frac{1}{2} \log \left( \frac{t + z}{t - z} \right) , \end{aligned}$$

while the latter is shown as a function of $r = \sqrt{x^2 + y^2}$. Note that the longitudinal variable is dimensionless while the transverse one is expressed in units of fermi (fm). Although formally unrelated, the dynamics of string fragmentation introduces a strong correlation between *y* and $y_{\tau }$, as illustrated in Fig. 14 for the default “middle” definition of production points. The spread from the diagonal comes from a number of effects, such as the probabilistic fragmentation process, given by Eq. (12), and hadronic decays.

**Fig. 15:**
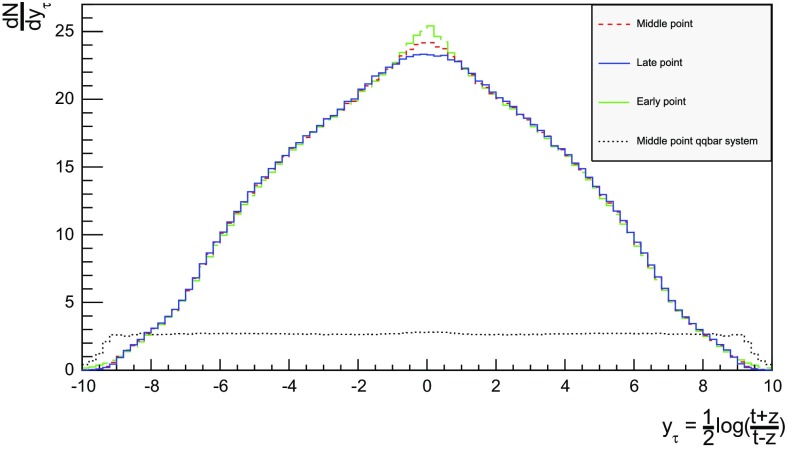
Longitudinal spectra for $\mathrm{p}\mathrm{p}$ events and $\mathrm{q}\bar{\mathrm{q}}$ systems, both at $\sqrt{s}=13$ TeV

**Fig. 16:**
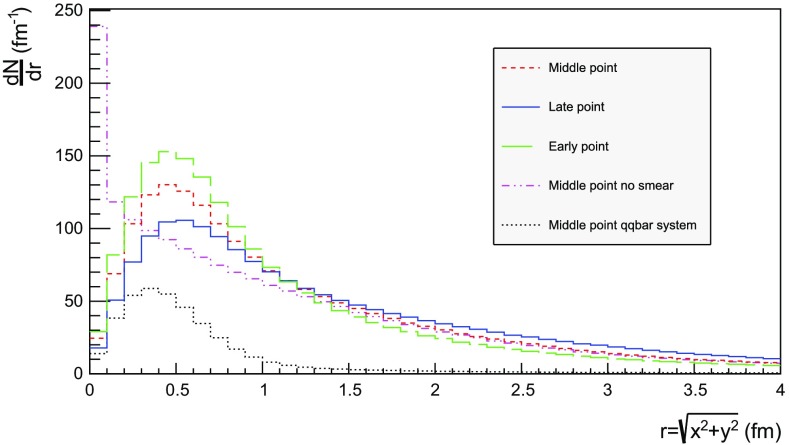
Transverse spectra for $\mathrm{p}\mathrm{p}$ events and $\mathrm{q}\bar{\mathrm{q}}$ systems, both at $\sqrt{s}=13$ TeV

Figures 15 and 16 display the longitudinal and transverse spectra for $\mathrm{p}\mathrm{p}$ collisions at $\sqrt{s} = 13$ TeV given by the “early”, “middle” and “late” definitions of hadron production points, represented in green, red and blue, respectively. In the same figures, the spectra for a single string, at the same CM energy, using the “middle” definition are also illustrated in black. Both primary and secondary hadrons are taken into account.

The longitudinal spectra for the different definitions are very similar, as can be seen in Fig. 15. The largest disagreement is visible around $y_{\tau } \approx 0$, where the spectra of the “early” definition peaks more, but “early” also has more particles at the very largest $y_{\tau }$ values. In short, the “early” alternative maximizes the extreme behaviour of hadron production, whereas the “late” one minimizes it. The differences are not bigger than that we can consider the “middle” definition a fairly reliable one.

Similar conclusions are drawn from the transverse spectra, shown in Fig. 16. The spectrum for $\mathrm{q}\bar{\mathrm{q}}$ events is a consequence of the transverse smearing, Sect. 3.4, and of particle decays; otherwise primary production would all be at $r=0$. In contrast, $\mathrm{p}\mathrm{p}$ events are constructed out of a large number of strings stretched between the partons from hard collisions, parton showers and beam remnants, all of them intrinsically with a transverse motion. Therefore the smearing is important for the spectrum at low *r* values, as can be seen in the difference between the two “middle” *r* distributions, while the distribution at larger *r* values is rather insensitive. The difference between the “early”, “middle” and “late” production points is larger than for the longitudinal spectra, but still sufficiently close as to give confidence that meaningful results can be obtained. In the following, all plots will be for the “middle” definition.

**Fig. 17:**
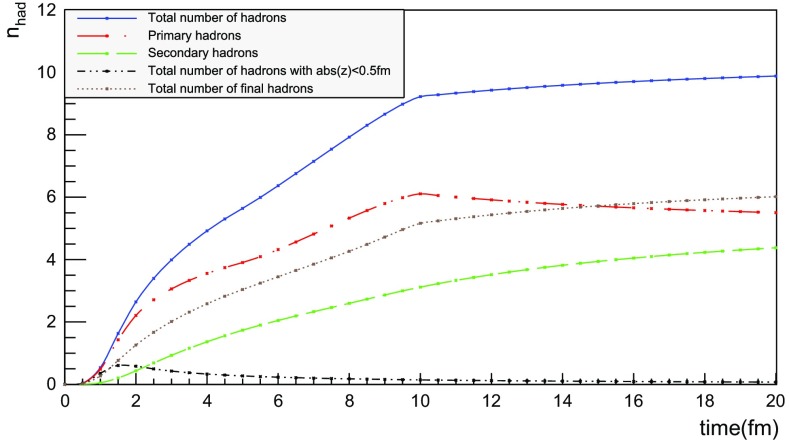
Hadron number per event as a function of time for a simple $\mathrm{q}\bar{\mathrm{q}}$ system formed by massless quarks in the CM frame with $\sqrt{s}=20$ GeV

### Temporal and radial evolution of hadron production

The number of hadrons is shown as a function of time for a single string with $\sqrt{s} = 20$ GeV in Fig. 17. The red curve corresponds to the number of primary hadrons, formed by the string fragmentation, that have not decayed at the time, while the green curve represents the number of secondary hadrons, from particle decays. The total number of hadrons, illustrated in blue, is the sum of primary and secondary hadrons. The brown curve represents the number of final (i.e. stable) hadrons, see Sect. 3.6. Finally, the black curve depicts the number of hadrons with $|z| < 0.5$ fm, to be discussed in Sect. 4.3.

For the 20 GeV simple $\mathrm{q}\bar{\mathrm{q}}$ system in its rest frame, the string can at most extend 10 fm in the $\pm z$ direction (for $\kappa = 1$ GeV/fm). This happens at $t = 10$ fm, since the massless quarks move with the speed of light. The primary hadron production therefore must stop at this time, as visible in Fig. 17. Decays make the number of hadrons continue to rise also beyond this time, but only slowly. Actually many hadrons, like the $\rho ^{\pm ,0}$ ones, are so short-lived that they decay within some fm of having been produced.

**Fig. 18:**
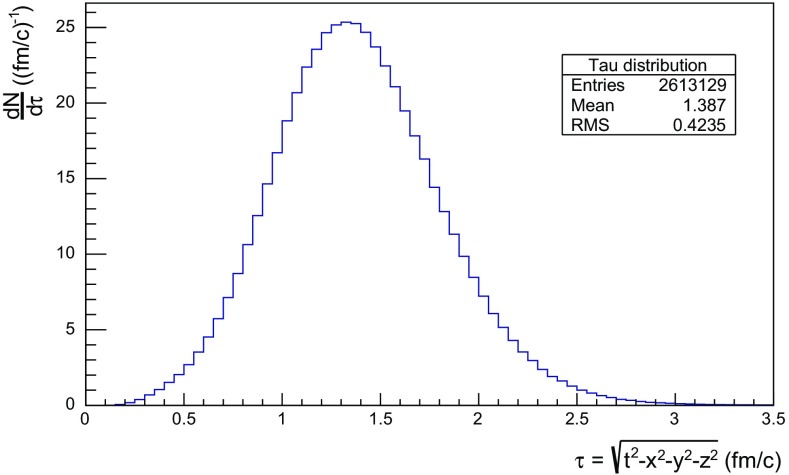
Invariant time $\tau$ distribution of primary hadrons in $\mathrm{q}\bar{\mathrm{q}}$ systems

Note that there are almost no hadrons in the system up until $t\approx 0.5$ fm, since the string has to have time to begin stretching out before it can begin to fragment. This is further illustrated in Fig. 18, with the invariant time distribution of primary hadron production points in the $\mathrm{q}\bar{\mathrm{q}}$ system. By default, the parameters *a* and *b* in Eqs. (12) and (14) are set to $a=0.68$ and $b=0.98$ GeV$^{-2}$ [41], giving rise to a suppression of small $\varGamma$ values of breakup vertices, and thereby also of small hadron production times. In detail, the relation between $\varGamma$ and $\tau$, Eq. (9), implies $P(\varGamma ) \propto \varGamma ^a \mathrm{d}\varGamma \propto \tau ^{2a} \, \tau \, \mathrm{d}\tau = \tau ^{2a+1} \, \mathrm{d}\tau$ for $\tau \rightarrow 0$. Furthermore, the expectation value of $\langle \varGamma \rangle = (1 + a)/b \approx 1.7$ GeV$^2$ gives $\langle \tau \rangle \approx \sqrt{ \langle \varGamma \rangle } /\kappa \approx 1.3$ fm, in agreement with Fig. 18. Because those aspects are typical of the fragmentation process, a similar behaviour is also observed in $\mathrm{p}\mathrm{p}$ collisions.

**Fig. 19:**
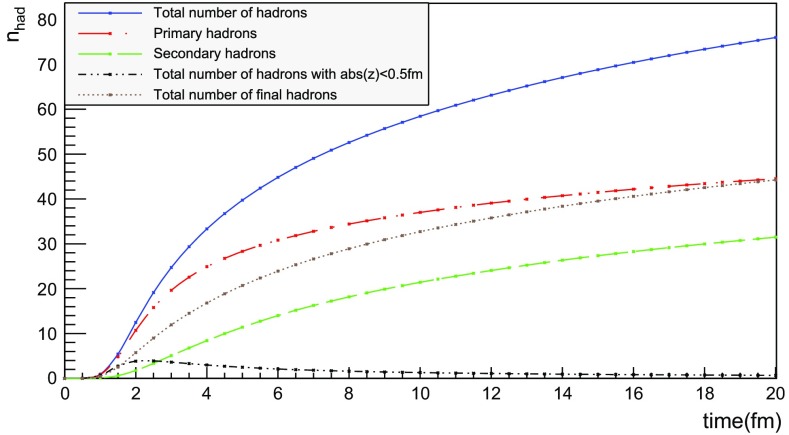
Hadron number per event as a function of time, up until $t = 20$ fm, for $\mathrm{p}\mathrm{p}$ collisions at $\sqrt{s}=13$ TeV

The time evolution of hadron production in 13 TeV $\mathrm{p}\mathrm{p}$ events is shown in Fig. 19 for $t \le 20$ fm. Although the qualitative behaviour is similar to the one in $\mathrm{q}\bar{\mathrm{q}}$ systems, the temporal evolution is smoother and the number of hadrons generated per unit time increases more rapidly in the $\mathrm{p}\mathrm{p}$ case. These effects are direct consequences of the presence of several string systems in $\mathrm{p}\mathrm{p}$ events, possibly extending all the way out to 6500 fm from the origin.

**Fig. 20:**
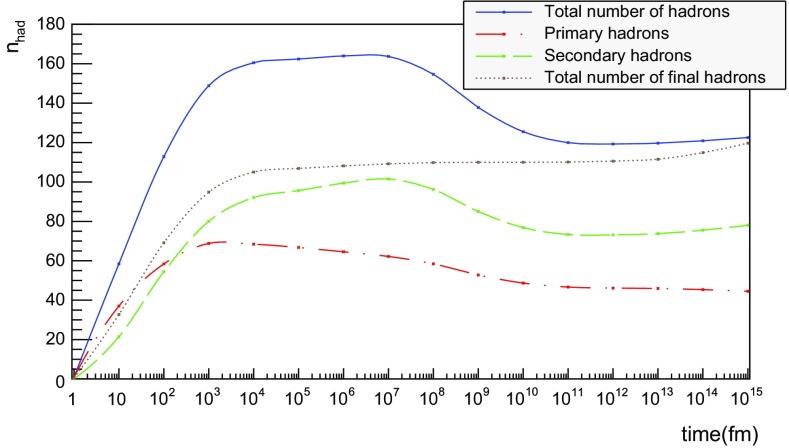
Hadron number per event as a function of time for 13 TeV $\mathrm{p}\mathrm{p}$ collisions

Figure 20 extends the $\mathrm{p}\mathrm{p}$ description up to $10^{15}$ fm $= 1$ m. As in the case of the $\mathrm{q}\bar{\mathrm{q}}$ system, the total number of primary hadrons increases until fragmentation is over, which now is at $t\approx 10^3$ fm owing to the higher energy. Decays deplete the number of remaining primary hadrons but increase the number of secondary ones. The significant drop in the number of hadrons at $t\approx 10^8$ fm is from electromagnetic decays of the $\pi ^0$, mainly $\pi ^0 \rightarrow \gamma \gamma$. Although the lifetimes of $\mathrm{s}$, $\mathrm{c}$ and $\mathrm{b}$ hadrons typically are at the mm to cm scales (more long-lived ones, like $\mathrm{K}^{\pm }$, being considered stable here), their decays are still ongoing at 1 m, owing to time dilation of the frequently fast-moving hadrons.

**Fig. 21:**
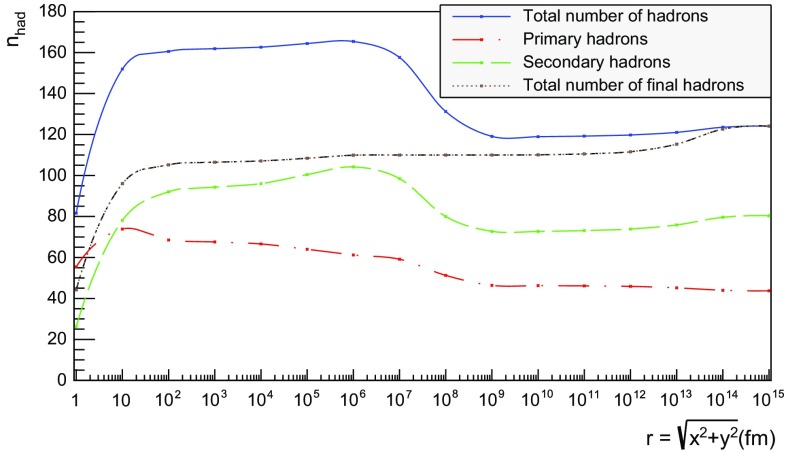
Hadron number per event as a function or radius for 13 TeV $\mathrm{p}\mathrm{p}$ collisions

Most of the expansion of the system is along the *z* axis, i.e. the |*z*| distribution of hadron production would look similar to the *t* one in Fig. 20, except for the lack of a suppression at $z = 0$. It is therefore interesting to show the radial evolution separately, Fig. 21, for the same *t* range. Overall the two figures resemble each other, but all the relevant features have been compressed owing to the lower radial velocities. The $\pi ^0 \rightarrow \gamma \gamma$ decay is shifted from $t\approx 10^8$ fm to $r \approx 10^6$ fm, for instance. The impact of weak $\mathrm{s}$, $\mathrm{c}$ and $\mathrm{b}$ hadron decays are better visible in the range between 1 and 100 mm; beyond that scale essentially all relevant decays have already occurred. At the other end of the scale, note that around half of the hadron production occurs in $r < 1$ fm; there is no equivalent dynamical suppression of small *r* as there is of small *t*.

**Fig. 22:**
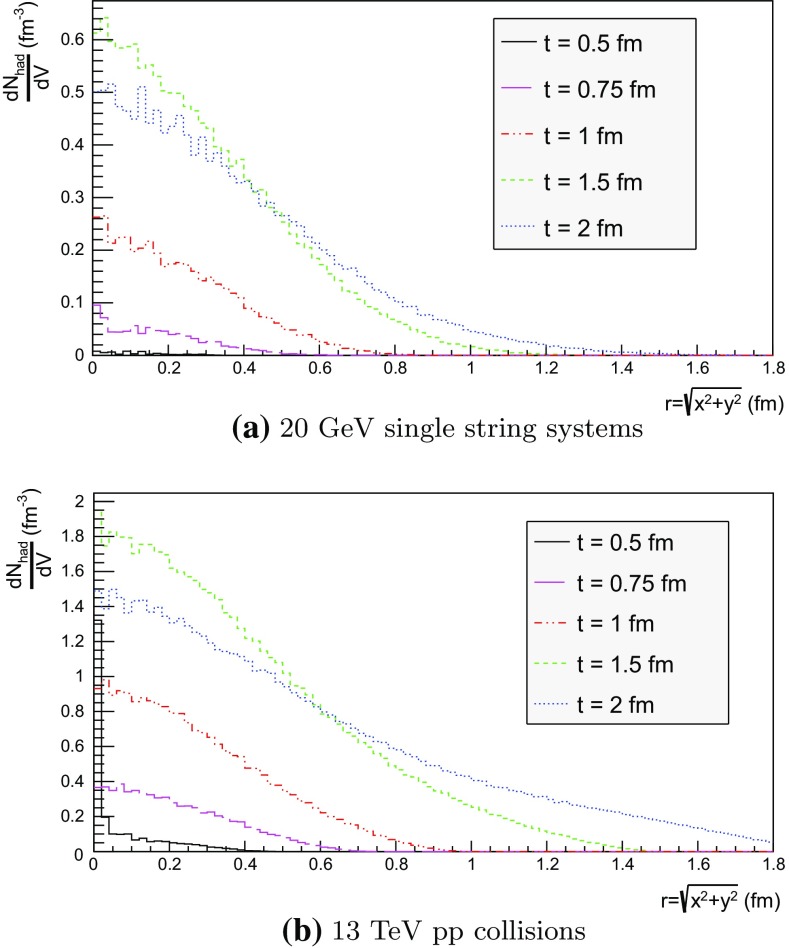
Hadronic density as a function of the radius for different constant times, for a central slice $|z| < 0.5$ fm

### Close-packing of hadron production in the central region

One of the key objectives of this article is to assess the space–time density of hadron production, $\mathrm{d}N/\mathrm{d}V$. Eventually we will need to use Lorentz invariant quantities, but these will then hide the time aspect of the evolution. To begin with, we will therefore study the density for $|z| \le 0.5$ fm as a function of *r* and *t*,

45 $$\begin{aligned} \left. \frac{\mathrm{d}N}{\mathrm{d}V} \right| _{|z| \le 0.5} = \left. \frac{\mathrm{d}N}{\mathrm{d}x \, \mathrm{d}y \, \mathrm{d}z} \right| _{|z| \le 0.5} = \frac{\mathrm{d}N}{\mathrm{d}x \, \mathrm{d}y} = \frac{\mathrm{d}N}{2\pi \, r \, \mathrm{d}r} , \end{aligned}$$

giving a measure of the hadronic densities as a function of radius. The *r*-integrated number as a function of *t* is shown in Figs. 17 and 19. This number only increases up to $t \approx 2$ fm, a time after which the longitudinal expansion leads to a steady decrease. Therefore, in Fig. 22a, the *r* distribution is only shown for a few different $t \le 2$ fm. The hadron density at times $t = 0.5$ fm is extremely low both for 20 GeV $\mathrm{q}\bar{\mathrm{q}}$ systems and for 13 TeV $\mathrm{p}\mathrm{p}$ events, since they hardly have had time to start hadronizing yet. From this point on, hadrons are generated from fragmentation and particle decays, giving an increasing hadron density in the central region. The maximal value is at $t \approx 1.5$ fm, a value that relates well with typical hadronization time scales, and where the density at $r = 0$ approaches 2 hadrons per fm$^3$. A proton has a volume $V_h = 4\pi r_{\mathrm{p}}^3/3 \approx 2.76$ fm$^3$ if we use $r_{\mathrm{p}}=0.87$ fm [35] so, assuming the same volume for all hadrons and disregarding potential Lorentz contraction effects, this implies that five hadrons overlap in the center of the collisions. That number increases rather slowly with the collision energy; it is around four hadrons at 2 TeV and seven at 100 TeV. Also other measures of close-packing are expected to display only a mild energy dependence, so our results at 13 TeV should offer guidance for a wide range of collider energies.

### Hadron production at different multiplicities

In order to extend the previous analysis to a Lorentz invariant measure of hadronic density, instead the volume element $\mathrm{d}^{3}x / t$ will now be used:

46 $$\begin{aligned} t \, \frac{\mathrm{d}N}{\mathrm{d}^3 x} = \frac{\mathrm{d}N}{\mathrm{d}^{2}r \frac{\mathrm{d}z}{t}} = \frac{\mathrm{d}N}{\pi \, \mathrm{d}r^2 \, \mathrm{d}y_{\tau }} \rightarrow \frac{N}{\pi \, r_m^2 \, \varDelta y_{\tau }} . \end{aligned}$$

In the last step $r_m$ is introduced as the median radius of the hadron creation vertices in the event and $\varDelta y_{\tau }$ is the full width at half maximum of the $\mathrm{d}N/\mathrm{d}y_{\tau }$ distribution. Together $r_m$ and $\varDelta y_{\tau }$ thus define a characteristic volume over which much of the production will occur, and relate it to a typical maximum density. For instance, the $|y_{\tau }|$ distribution is roughly triangular in shape, cf. Fig. 15, so $N/\varDelta y_{\tau }$ is about the height of the $\mathrm{d}N/\mathrm{d}y_{\tau }$ distribution at its maximum.

Note that the hadronic multiplicity studied here is different from typical experimental definitions, e.g. the charged multiplicity in vertex detectors. Since we are interested in the hadronization process, only strong decays should be taken into account in our analysis. This excludes electromagnetic and weak decays, such as the $\pi ^0$ one, but furthermore decays with $r > 10$ fm are not taken into account, since beyond that hadronic densities have fallen to modest levels anyway. In order to avoid double-counting of a hadron and its decay products, all secondary hadronic decay vertices enter with a weight one less than the hadronic multiplicity of the decay. Counted this way, the average multiplicity of inelastic nondiffractive 13 TeV $\mathrm{p}\mathrm{p}$ events is $n_{\mathrm {had}} = 169$.

**Fig. 23:**
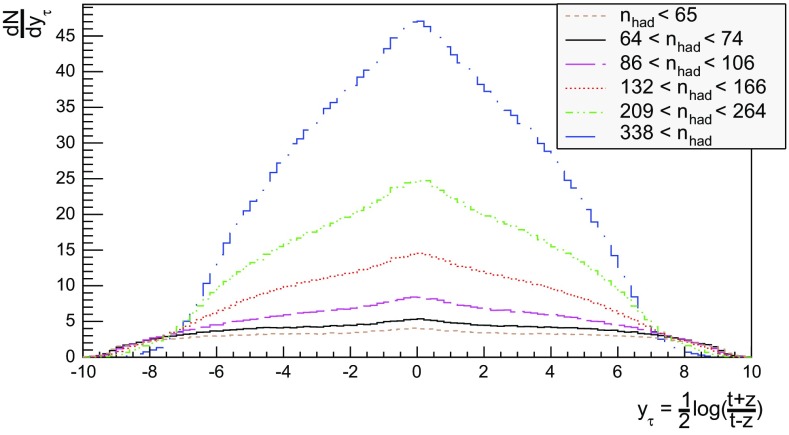
Longitudinal spectra for 13 TeV $\mathrm{p}\mathrm{p}$ collisions and different multiplicity ranges

**Fig. 24:**
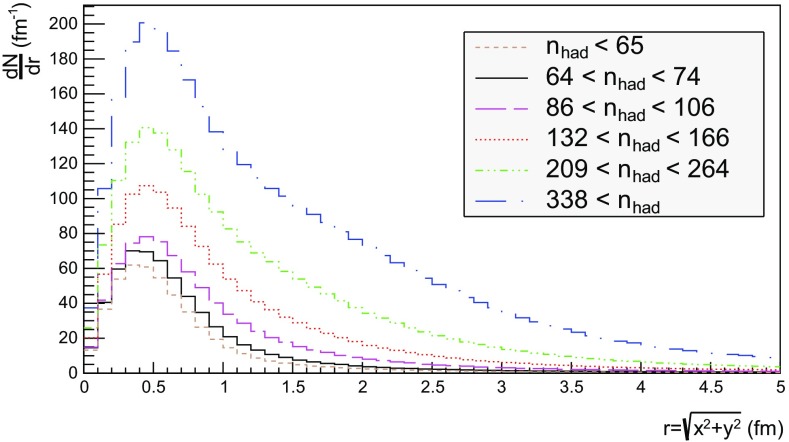
Transverse spectra for 13 TeV $\mathrm{p}\mathrm{p}$ collisions and different multiplicity ranges

Inside this sample, ten multiplicity ranges are defined such that each of them corresponds to roughly 10% of the events. The resulting longitudinal $y_{\tau }$ and transverse *r* spectra are presented in Figs. 23 and 24, respectively. For the sake of clarity, some intermediate multiplicity bins are left out of the figures. By energy–momentum conservation the $y_{\tau }$ (and *y*) spectra are more peaked around the middle for increasing multiplicities. Not so for the *r* spectra, where the distribution shifts towards larger values for the higher multiplicities. It is here useful to remind that the basic MPI framework implies that high multiplicities primarily come from having more MPIs, rather than e.g. from a single hard interaction at a larger $p_{\perp }$ scale, and that therefore $\langle p_{\perp }\rangle (n_{\mathrm {charged}})$ is expected to be reasonably flat. The experimental observation of a rising $\langle p_{\perp }\rangle (n_{\mathrm {charged}})$ actually was the reason to introduce colour reconnection (CR) as a key part of a realistic MPI modelling [2].

**Fig. 25:**
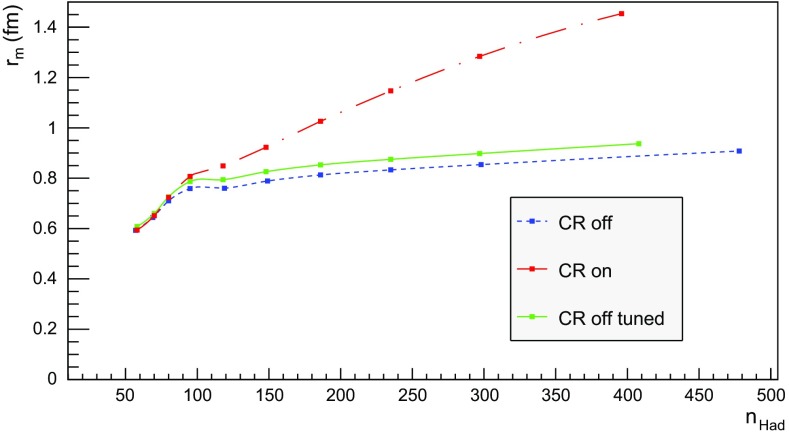
Median radii as a function of multiplicity for 13 TeV $\mathrm{p}\mathrm{p}$ collisions. The red curve corresponds to the approach with colour reconnection, while the blue and green curves represent the model without colour reconnection and a tuned model without colour reconnection, respectively

The effect of CR on the median radii $r_m$ is shown in Fig. 25, as a function of the median hadronic multiplicity $n_{\mathrm {had}}$ of each multiplicity range. The red and blue curves represent results with and without CR, respectively, and these match very well with expectations from the $\langle p_{\perp }\rangle (n_{\mathrm {charged}})$ behaviour; also the rise of $r_m$ is driven by the CR mechanism. Note that switching off CR gives higher event multiplicities, well above data. To this end also a green curve is introduced, wherein the $p_{\perp 0}$ parameter of the MPI framework [3] is increased for the no-CR alternative until the average multiplicity is the same as in the default with-CR scenario. This gives a slightly larger $r_m$ than the naive no-CR setup, since the $\langle p_{\perp }\rangle$ of MPIs is increased in the process, but otherwise is in line with the original observation.

**Fig. 26:**
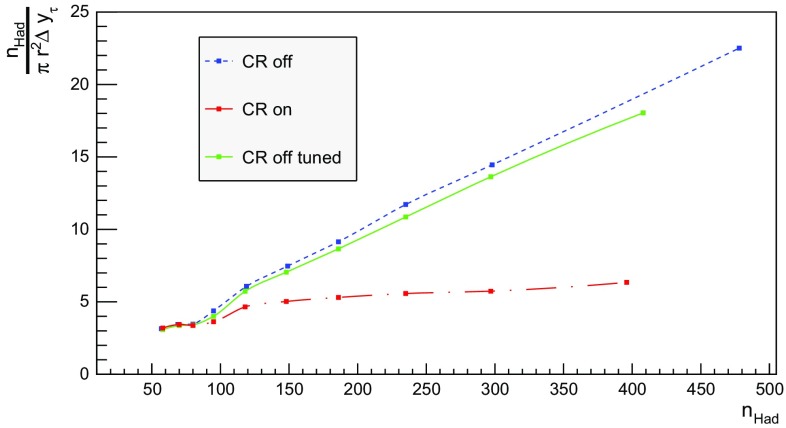
Hadron density as a function of multiplicity for $\mathrm{p}\mathrm{p}$ collisions at 13 TeV. The red, blue and green curves represent the three different models with and without colour reconnection, also included in Fig. 25

Figure 26 shows the hadron density, defined as in Eq. (46), for the three same scenarios as above. The $n_{\mathrm {had}}$, $r_m$ and $\varDelta y_\tau$ are calculated in each multiplicity range. The space–time hadron density increases with hadronic multiplicity, but significantly faster in the two scenarios without CR, as a direct consequence of the inverse quadratic dependence on $r_m$. The lower values with CR on may be partly misleading, however; only because strings are spread across a bigger transverse area when CR is on, it does not mean that there are strings everywhere in that area. The typical average density of 5 hadrons per Lorentz invariant space–time element should therefore be viewed as a lower estimate.

### Close-packing analysis in the hadron rest frame

As a final measure of close-packing we will next check how many hadrons overlap with each of the hadrons of an event, as defined in the rest frame of the hadron at the time when it is formed. In detail, consider a hadron $h_1$ generated at time $t_1$, where $t_1$ is defined in the rest frame of hadron $h_1$. The other hadrons in the system are boosted to the rest frame of $h_1$, where only the hadrons created at times $t \le t_1$ and which have not decayed at $t_1$ are taken into account. Their location at $t_1$ is calculated from the respective production point and four-momentum, from which the distance to $h_1$ can be calculated. If this distance is shorter than $2r_{\mathrm{p}}$, $r_{\mathrm{p}}$ being the proton radius, the hadrons are considered to overlap, implying that already the production of $h_1$ could be affected by the presence of these other hadrons. Note that Lorentz contraction is not taken into account, which would decrease numbers, but then neither is the possibility of closer distances at $t > t_1$, which would increase them. The analysis is done including or excluding the adjacent hadron on each side along the string of the hadron studied. The reason for the latter scenario is that any effects of same-string-neighbours already effectively should have been taken into account in the tuning of the fragmentation process, e.g. in Eq. (12).

**Fig. 27:**
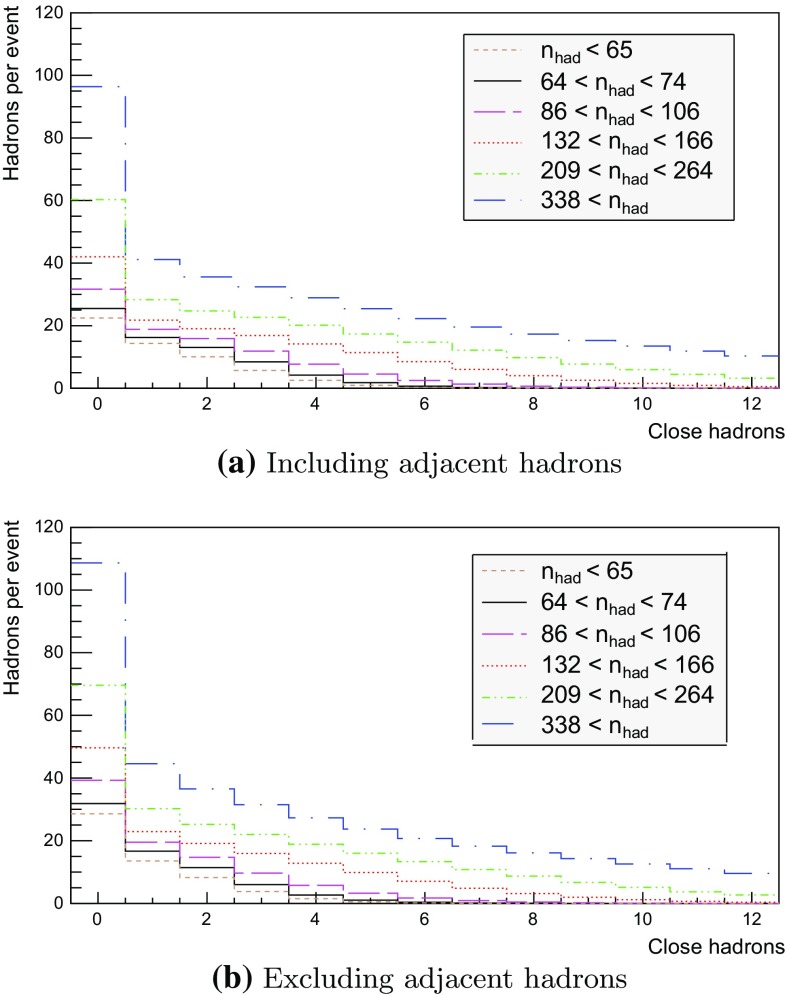
Hadron overlap for different multiplicity ranges for 13 TeV $\mathrm{p}\mathrm{p}$ collisions

The number of overlapping hadrons is shown in Fig. 27 for different hadronic multiplicity ranges, as presented in Sect. 4.4. Although close-packing also takes place in low-multiplicity $\mathrm{p}\mathrm{p}$ events, the number of hadrons overlapping with a newly created one is not so high. For high-multiplicity events, on the other hand, close-packing often arises with a significant number of nearby hadrons, likely leading to collective effects that are not taken into account in Pythia.

**Fig. 28:**
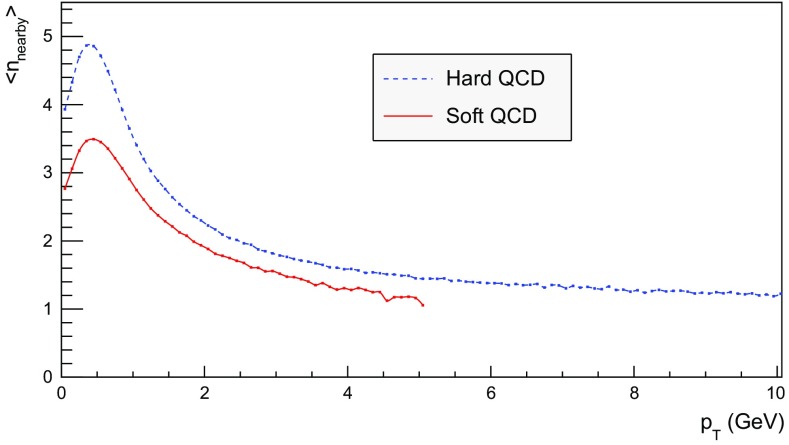
Average number of overlapping hadrons as a function of the $p_{\perp }$ of the hadron studied. The red and blue distributions illustrate the soft and hard QCD processes, where the former stops at 5 GeV owing to limited statistics at large $p_{\perp }$

The overlap can be differentiated further. Generally, particles produced at large transverse momenta are not expected to experience close-packing as much as those at small ones. The reason is that, even if parton showers can generate many partons from each initial high-$p_{\perp }$ parton, these daughter partons are spread widely in momentum space. Therefore, the fragmenting strings stretched between them also will have a modest overlap, unlike the accretion of low-$p_{\perp }$ strings from multiple soft MPIs. In order to isolate this feature, we study the overlap as a function of the hadron transverse momentum, using the same analysis procedure as above, with exclusion of adjacent hadrons along the string, Fig. 28, for “soft” and “hard” QCD events in red and blue, respectively. The former is the standard inelastic nondiffractive event sample, whereas the latter is for the subsample where a hard $2 \rightarrow 2$ QCD process has $p_{\perp }> 100$ GeV. In both cases the overlap peaks for hadrons around $p_{\perp }\approx 0.5$ GeV, and then falls off at larger $p_{\perp }$ values. The level is somewhat higher for the hard-QCD events, consistent with such events being biased towards smaller impact parameters and therefore more MPIs, but the trends are consistent.

## Summary and outlook

The motivation for this article is the mounting evidence for several collective effects in high-multiplicity $\mathrm{p}\mathrm{p}$ collisions, similar to those usually associated with the formation of a Quark–Gluon Plasma in heavy-ion collisions. Whether we are witnessing QGP also in $\mathrm{p}\mathrm{p}$ or not remains an open question, but the need to allow for some kind of collective mechanisms can hardly be in doubt. It should not even come as a surprise, given that already order-of-magnitude estimates of the size of the fragmentation region told us that strings would be formed close-packed and fragment into close-packed hadrons within any realistic MPI-based scenario. Colour reconnection was introduced as a partonic-state mechanism to describe some signals of collectivity, notably the rise of $\langle p_{\perp }\rangle (n_{\mathrm {charged}})$. But the rising fraction of multistrange baryon production with event multiplicity implies that collective effects are needed also in or after the fragmentation stage, or both.

To be able fully to explore various such scenarios it becomes important to understand the space–time structure of hadronization in more detail than hitherto. The aim of this article has been to develop the necessary framework, and implement it as part of the public Pythia event generator. Specifically, we have determined the space–time location of the string breakup vertices and compared three alternative definitions for primary hadron production points. Although the implementation of the space–time picture in a simple $\mathrm{q}\bar{\mathrm{q}}$ string topology is straightforward, the picture gets much more intricate when more complicated topologies are addressed.

To illustrate the usefulness of the new framework, some simple first studies have been presented, notably exploring space–time hadron densities. Initially, inclusive longitudinal and transverse space-time distributions were shown, and the production and decay patterns from fm to m scales were traced. Next the density in a central slice $|z| < 0.5$ was studied as a function of *t* and *r*. While not explicitly Lorentz invariant, it gave some first hints of close-packing problems. Moving from a volume element $\mathrm{d}^3 x$ to $\mathrm{d}^3 x/t$ gave access to Lorentz-invariant density measures. It was shown that the median radius of the fragmentation region is increasing with multiplicity, but almost only because of the colour reconnection effects. The flip side is that density is increasing significantly with multiplicity without CR, whereas it remains at an average of about five hadrons overlapping with CR included.

The close-packing of hadrons was finally analysed by counting the number of hadrons overlapping with a newly generated one in its rest frame, again for different event multiplicities. In this case, the number of nearby hadrons does increase with multiplicity, with CR included, implying that close-packing becomes increasingly important with multiplicity also here. The overlap is largest for low-$p_{\perp }$ hadrons, in the MPI-dominated region, whereas it drops for larger $p_{\perp }$ scales, dominated by hard QCD jets.

A few corners have been cut in the current $\mathrm{p}\mathrm{p}$ implementation. Notably no space–time vertices have been assigned to the individual MPI collisions, although such assignments are implicit in the MPI impact-parameter and matter-profile framework [3]. A sensible space–time picture of parton-shower evolution would introduce offsets, although presumably not major ones. Similarly, the CR between different MPIs implies that the two ends of a string may start out from different space–time points. For now, all such effects have implicitly been made part of the generic smearing step in Sect. 3.4.

To these minor corrections should be added the potentially much larger dynamical ones that could generate collective effects, be it before, during or after the string fragmentation stage. The shove and rope mechanisms are two examples for the first two stages, but the immediate continuation of the current article would be to study the consequences of hadronic rescattering in a dense hadronic gas. Models for hadronic rescattering already exist [42], such as UrQMD [43] and SMASH [44], and could possibly be interfaced. For better control, however, it would be useful to implement relevant aspects of such a framework as an integrated part of the Pythia program.

The longer-term expectation is that continued experimental studies will provide further information on all kinds of collective phenomena in LHC $\mathrm{p}\mathrm{p}$ events, and that model building will try to rise to the challenge. Especially interesting is to figure out which phenomena can be explained without invoking QGP, and which cannot. This would then reflect back on the LHC heavy-ion program.

## Figures and Tables

**Fig. 29:**
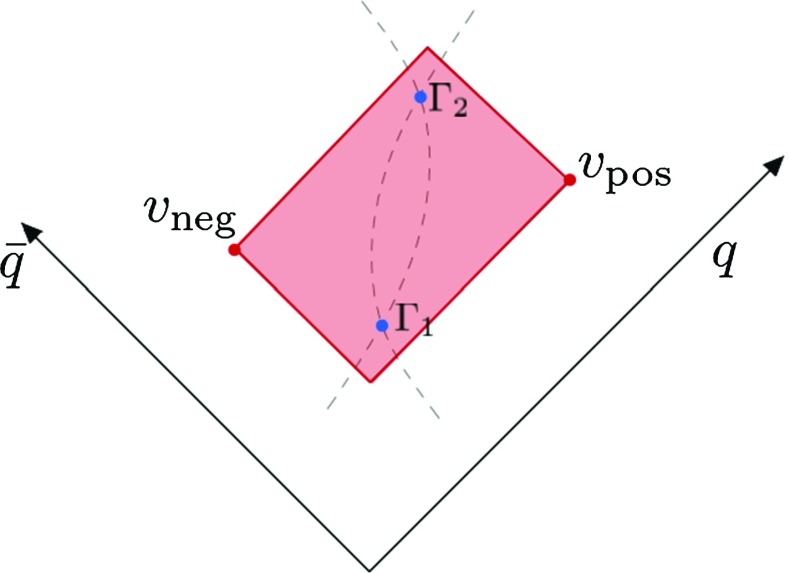
The two possibilities for the final breakup, in blue, and the final region, in red, in a $\mathrm{q}\bar{\mathrm{q}}$ system. The two old breakups, $v_{\mathrm {neg}}$ and $v_{\mathrm {pos}}$, are also represented in red

**Fig. 30:**
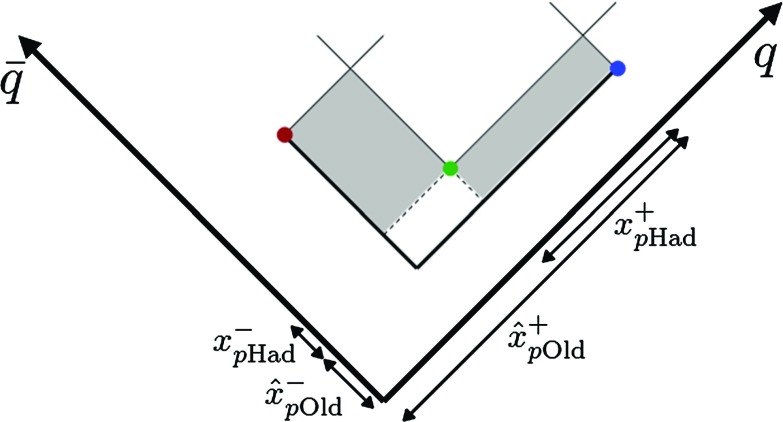
Final breakup point and final two hadrons, corresponding to the grey regions, in a $\mathrm{q}\bar{\mathrm{q}}$ system. The red and blue points are the previous breakups and the endpoints of the final region, while the green dots represents the final breakup

**Fig. 31:**
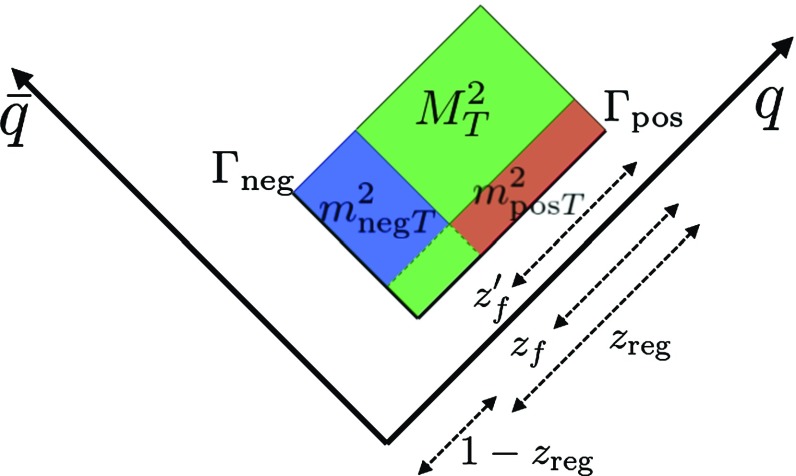
Final breakup and final two hadrons of a $\mathrm{q}\bar{\mathrm{q}}$ system. The blue and red areas represent the two final hadrons while the green area corresponds to the final region

**Fig. 32:**
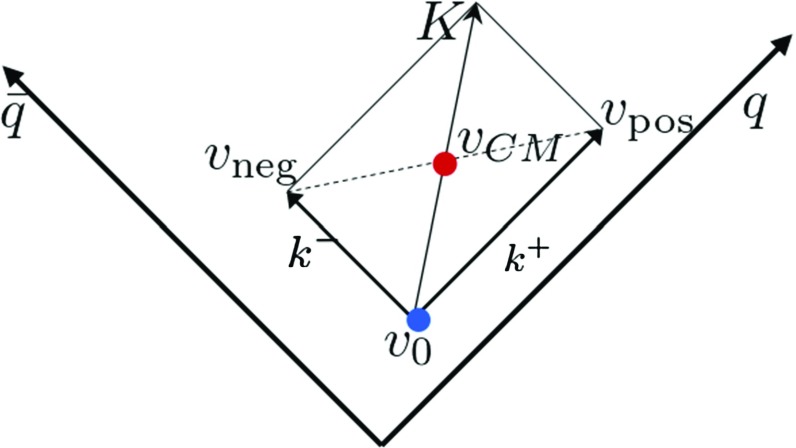
Final region of a $\mathrm{q}\bar{\mathrm{q}}$ system. The origin of the region is represented by $v_0$ while $v_{\mathrm {CM}}$ stands for the location of the CM of the region. The four-momentum of the final region is defined as *K*. As in other cases, $v_{\mathrm {pos}}$ and $v_{\mathrm {neg}}$ represent the old breakups

## Appendix A: Space–time location of the final breakup

Fragmentation of an open string is modelled by allowing hadron production from either string end, until the remaining invariant mass of the system is only sufficient to generate the last two hadrons (see Sect. 3.1). At that point, a final breakup is generated between the last previous breakup on either side. For the energy–momentum picture, the final breakup occurs in a fictitious final region, created from the combination of all the unused parts of all remaining regions. This region does not have a space–time correspondence, however; in particular there is no concept of a space–time offset where this region is created. In this case we therefore have had to depart from the energy–momentum picture, to develop an unfortunately rather complex procedure.

To set the stage, consider a simple $\mathrm{q}\bar{\mathrm{q}}$ string, where the flavours of the final breakup helps define the two final hadrons. The transverse mass constraint of each of those hadrons is represented by hyperbolae, see Sect. 2.2.3. The two hyperbolae either do not cross at all or else cross in two different points. No solution can be found in the former case, and the fragmentation process then has to be repeated. The latter case is illustrated in Fig. 29, where the remaining region is depicted in red and the blue dots represent the two points where the hyperbolae meet. Since the two possibilities have different $\varGamma$ values, the relative probabilities for them to occur are given by Eq. (14). For simplicity, only the exponential part is retained, i.e. $P(\varGamma _i) \propto \exp (-b\varGamma _i)$ is used to pick either point. That choice made, the kinematics is fully defined.

Major complications are found in systems with several intermediate gluons between the $\mathrm{q}$ and $\bar{\mathrm{q}}$ ends, specifically when the two old breakups are located in different regions. In those cases, knowing the region in which the final breakup is located is not always possible. Since that aspect is essential to calculate the $\hat{x}^{\pm }$ fractions, several methods have been tested before settling on the one presented here.

For notational convenience, the old breakup closer to the $\mathrm{q}$ ($\bar{\mathrm{q}}$) end will be called the positive (negative) breakup, at location $v_{\mathrm {pos}}$ ($v_{\mathrm {neg}}$) in the positive (negative) region, together with the final breakup forming the positive (negative) hadron. The first implemented method consists in projecting the positive/negative hadron four-momentum on to the longitudinal and transverse direction vectors of the positive/negative region [27]. If the two old breakups are in the same region then that is also the region of the final vertex. This situation is exemplified in Fig. 30, where the green point is the final breakup, the red and blue points are the old breakups, which correspond to the endpoints of the final region, and the grey squares represent the two final hadrons created. As can be seen in the figure, the $x^{\pm }$ fraction of the positive hadron are $x_{p,\mathrm {Had}}^{\pm }$ and the $\hat{x}^{\pm }$ of the positive breakup are $\hat{x}_{p,\mathrm {Old}}^{\pm }$. Then, the $\hat{x}_f^{\pm }$ of the final breakup can be obtained as,

47 $$\begin{aligned}&\hat{x}_f^+ = \hat{x}_{p,\mathrm {Old}}^+ - x_{p,\mathrm {Had}}^+ ,\nonumber \\&\hat{x}_f^- = \hat{x}_{p,\mathrm {Old}}^- + x_{p,\mathrm {Had}}^- . \end{aligned}$$

The same procedure can be followed with the negative breakup and the negative hadron, giving,

48 $$\begin{aligned}&\hat{x}_f^+ = \hat{x}_{n,\mathrm {Old}}^+ + x_{n,\mathrm {Had}}^+ ,\nonumber \\&\hat{x}_f^- = \hat{x}_{n,\mathrm {Old}}^- - x_{n,\mathrm {Had}}^- . \end{aligned}$$

In the general case, the solutions of Eqs. (47) and (48) will agree only if the positive and negative regions coincide. But the projection method can also be used when the positive and negative regions are different, in which case the longitudinal momentum of the positive or negative hadron is projected on the corresponding region, using either Eq. (47) or Eq. (48). If either of these give projected values $0 \le \hat{x}^{\pm }_f \le 1$ then a solution has been found in the respective region, and we are done. If not, the search continues.

One of the main complications to obtaining the $\hat{x}^{\pm }_f$ values is that the *z* value of the final breakup is not calculated, since it is not needed in the energy-momentum picture. If the projection method fails, the *z* value can be calculated from the $\varGamma$ of the old breakups and the transverse mass of the final region, i.e. of the final two hadrons combined. These variables are depicted in Fig. 31, along with $z_f$ and $z'_f$, which correspond to the $z^+$ fractions of the positive hadron in the real region and the $z^+$ fraction with respect to the final region, i.e, when the $\tilde{p}^+$ and $\tilde{p}^-$ of the final region are normalized to unity. The $z_{\mathrm {reg}}$ variable in the figure represents the $z^+$ fraction taken from the negative breakup if the final breakup was not created. This value can be calculated from the relation given by Eq. (13), which in this case gives,

49 $$\begin{aligned} \varGamma _{\mathrm {neg}} = (1-z_{\mathrm {reg}}) \left( \varGamma _{\mathrm {pos}} + \frac{M_{\perp }^2}{z_{\mathrm {reg}}}\right) , \end{aligned}$$

where the final breakup was not taken into account. From this relation, the value of $z_{\mathrm {reg}}$ is found to be,

50 $$\begin{aligned} z_{\mathrm {reg}} = \frac{\sqrt{X^2 + 4 M_{\perp }^2 \varGamma _{\mathrm {pos}}} - X}{2\varGamma _{\mathrm {pos}}} ,~~ X = M_{\perp }^2 + \varGamma _{\mathrm {neg}} - \varGamma _{\mathrm {pos}}.\nonumber \\ \end{aligned}$$

During the fragmentation process in Pythia only the fractions $z'_f$ are determined by considering $z_{\mathrm {reg}} = 1$, as stated previously. Hence, the $z_f = z_f^+$ fractions can be calculated from $z'_f$ and $z_{\mathrm {reg}}$ using the relation $z_f = z'_f \ z_{\mathrm {reg}}$. Note that $z_f = z'_f$ if $z_{\mathrm {reg}} = 1$, as expected. The same process can be followed to calculate $z_f^-$, swapping the variables $\varGamma _{\mathrm {pos}}$ and $\varGamma _{\mathrm {neg}}$. Once the $z_f$ are known, the $\hat{x}^{\pm }$ fractions of the final breakup can be determined from Eq. (11) and its space–time location deduced as in Sect. 3.

Although the last procedure succeeds in the large majority of cases, sometimes the region of the final breakup cannot be found. Then the location of the final breakup is calculated from the old breakups by determining a space–time location of the origin of the artificial final region used in the energy–momentum picture. As can be deduced from Fig. 32, the space–time location of the CM of the final region is defined by $v_{\mathrm {CM}} = (v_{\mathrm {pos}} + v_{\mathrm {neg}}) /2$. This final region can be treated as a $\mathrm{q}\bar{\mathrm{q}}$ system with four-momentum $K = k^+ + k^-$, where $k^{\pm }$ are the four-momenta vectors of the two endpoints. Following the same approach as used to derive the early hadron production point (see Sect. 3.1), the space–time location of origin of the final region is given by,

51 $$\begin{aligned} v_0 = v_{\mathrm {CM}} - \frac{1}{2} K. \end{aligned}$$

Considering $\hat{x}'^{\pm }_f$ to be the $\hat{x}^{\pm }$ fractions of the final breakup in the final region, the space–time location of the final breakup can then be calculated as,

52 $$\begin{aligned} v_f= & {} v_0 + \hat{x}'^+ k^+ + \hat{x}'^- k^- \nonumber \\= & {} v_{\mathrm {CM}} + \left( \hat{x}'^+ - \frac{1}{2}\right) k^+ + \left( \hat{x}'^- - \frac{1}{2}\right) k^- . \end{aligned}$$

Eq. (52) holds in the transverse rest frame, when the final region system is not evolving in time. That is not the case if the two old breakups are in different regions. In order to find an expression valid for all the cases, we define the variable $r = \sqrt{l^2/K^2}$, with $l^2 = - (v_{\mathrm {neg}} - v_{\mathrm {pos}})^2$, to quantify how much the final string system differs from the system in the transverse rest frame. Then, the origin of the final region in the string system is determined by $v_0 = v_{\mathrm {CM}} - Kr/2$ and the general expression for the space–time location of the final breakup is

53 $$\begin{aligned} v_f = v_{\mathrm {CM}} + \left( \hat{x}'^+ - \frac{r}{2}\right) k^+ + \left( \hat{x}'^- - \frac{r}{2}\right) k^- . \end{aligned}$$

The methods presented in this section are implemented in Pythia to obtain the space–time location of the final breakup. First the projection method is executed from the $\mathrm{q}$ or positive side. If it fails, the same method from the negative side is carried out. Whenever the projection method fails, the $z^+$ value is calculated. In case of failure with this, the same method is carried out to calculate $z^-$. If none of the previous methods work, the space–time location of the final breakup is determined by Eq. (53). The different procedures are carried out in order of decreasing accuracy.

## Appendix B: Correction to non-physical situations

As mentioned in Sect. 2.2.3, by definition $0< \hat{x}^{\pm } < 1$. Although this should always be the case, Pythia allows values outside that range whenever a step is taken from one region to a new one. Since the $\hat{x}^{\pm }$ fractions were only used to determine the energy and momentum of the hadrons, stepping outside the allowed range was not a problem, as long as the hadron energy was positive. Nevertheless, fractions outside the $0< \hat{x}^{\pm } < 1$ range are a significant problem in the space–time picture, since they can lead to negative times or negative squared invariant times of the breakup locations. In order to address these unphysical situations, one of two corrections is applied in the space–time implementation, before adding the region offset, the smearing in transverse space and the massive correction.

The first option consists in adjusting the old space–time location of the breakup by a fraction of the region four-momentum, such that the new squared invariant time is equal to zero. Then, the expression of the new breakup location is determined by

54 $$\begin{aligned} v_{\mathrm {new}} = v_{\mathrm {old}} + \xi p_{\mathrm {reg}} , \end{aligned}$$

where $\xi$ is found by requiring $v_{\mathrm {new}}^2 = 0$.

In the second option, $\hat{x}^{\pm }$ fractions outside the $0< \hat{x}^{\pm } < 1$ are set to the value at the closest border, i.e. 0 or 1, and the space–time location is recalculated according to Eq. (23).

The option adopted is the one that gives the smallest change of the space–time breakup location.

## Footnotes

1 After posting the preprint, we learned of another related implementation [28]. It does not address all the issues considered here, however, and therefore is insufficient e.g. for LHC studies.
